# Mesenchymal Cells Support the Oncogenicity and Therapeutic Response of the Hedgehog Pathway in Triple-Negative Breast Cancer

**DOI:** 10.3390/cancers11101522

**Published:** 2019-10-10

**Authors:** Ana M. Reyes-Ramos, Karla P. Ramos-Cruz, Nelson J. Rodríguez-Merced, Michelle M. Martínez-Montemayor, Nelson D. Franqui-Ríos, Jan P. Ríos-Grant, Andrea Flores, Gerónimo Maldonado-Martínez, Wandaliz Torres-García, Maribella Domenech

**Affiliations:** 1 Department of Chemical Engineering, Universidad de Puerto Rico-Mayagüez, Mayagüez, PR 00680, USA; anamilena.reyes@upr.edu (A.M.R.-R.); karlapr2@illinois.edu (K.P.R.-C.); nelson.rodriguez20@upr.edu (N.J.R.-M.); 2 Department of Biochemistry, Universidad Central del Caribe, School of Medicine-Bayamón, Bayamón, PR 00956, USA; michelle.martinez@uccaribe.edu; 3 Industrial Biotechnology Program, Universidad de Puerto Rico-Mayagüez, Mayagüez, PR 00680, USA; nelson.franqui@upr.edu (N.D.F.-R.); jan.rios2@upr.edu (J.P.R.-G.); andrea.flores2@upr.edu (A.F.); 4 Data Management and Statistical Research Support Unit, Universidad Central del Caribe, School of Medicine-Bayamón, Bayamón, PR 00956, USA; geronimo.maldonado@uccaribe.edu; 5 School of Chiropractic, Universidad Central del Caribe, School of Medicine-Bayamón, Bayamón, PR 00956, USA; 6 Department of Industrial Engineering, Universidad de Puerto Rico-Mayagüez, Mayagüez, PR 00680, USA; wandaliz.torres@upr.edu

**Keywords:** Hedgehog signaling, triple-negative breast cancer, CAFs, EMT, mesenchyme, tumor microenvironment

## Abstract

The paracrine interaction between tumor cells and adjacent stroma has been associated with the oncogenic activity of the Hedgehog (Hh) pathway in triple-negative breast tumors. The present study developed a model of paracrine Hh signaling and examined the impact of mesenchymal cell sources and culture modalities in the oncogenicity of the Hh pathway in breast tumor cells. Studies consisted of tumor cell monocultures and co-cultures with cancer-associated and normal fibroblasts, tumor cells that undergo epithelial–mesenchymal transition (EMT), or adipose-derived mesenchymal stem cells (ADMSCs). Hh ligand and pathway inhibitors, GANT61 and NVP-LDE225 (NVP), were evaluated in both cell cultures and a mouse xenograft model. Results in monocultures show that tumor cell viability and Hh transcriptional activity were not affected by Hh inhibitors. In co-cultures, down-regulation of *GLI1*, *SMO*, and *PTCH1* in the stroma correlated with reduced tumor growth rates in xenografted tumors and cell cultures, confirming a paracrine interaction. Fibroblasts and EMT cells supported Hh transcriptional activity and enhanced tumor cell growth. Mixed and adjacent culture modalities indicate that tumor growth is supported via fibroblast-secreted soluble factors, whereas enriched tumor stemness requires close proximity between tumor and fibroblasts. Overall this study provides a tumor–mesenchymal model of Hh signaling and highlights the therapeutic value of mesenchymal cells in the oncogenic activity of the Hh pathway.

## 1. Introduction

Hedgehog (Hh) signaling regulates epithelial–stromal interactions during tissue development [1], and is reactivated in adult tissues in response to injury [1,2,3]. Canonical Hh signaling consists of the Sonic hedgehog (SHH)-ligand binding to the transmembrane receptor Patched1 (PTCH1), an action that relieves repression of another transmembrane protein, Smoothened (SMO). SMO triggers downstream signal transduction that includes the activation of transcription factors glioma associated oncogene family zinc finger 1(GLI1) and glioma associated oncogene family zinc finger 2 (GLI2) through the dissociation of suppressor of fused homolog (SUFU) and down-regulation of the transcriptional repressor glioma-associated oncogene family zinc finger 3(GLI3). Sustained and uncontrolled Hh activation has been associated with tumor initiation and progression in several cancers of pancreas [4], colon [5], prostate [6], lung [7,8], and breast tissues [9,10].

High levels of expression of several of the Hh pathway components have been identified in 30% to 40% of diagnosed cases [11,12] and correlated with reduced survival rates in patients of the triple-negative breast cancer (TNBC) subtype [9,12,13,14,15,16]. Several studies indicate that the tumor-adjacent stroma is a primary therapeutic target of Hh inhibitors in TNBC [17,18]. This paracrine mechanism is characterized by binding of tumor-secreted Hh ligands (SHH, Indian Hedgehog (IHH), or Desert Hedgehog (DHH)) to PTCH1 receptors, and elevated levels of GLI1, GLI2, PTCH1, and SMO genes in the tumor-adjacent stroma. A study by Valenti et al. showed that treatment of tumors with a Hh inhibitor promoted the delay of tumor formation, which correlated with a reduced amount of mouse fibroblasts and limited expansion of cancer stem cells in basal-like mammary gland tumors [19]. In another TNBC mouse model, genomic profiling of cell populations further confirmed that this paracrine interaction is restricted to cancer-associated fibroblasts (CAFs) and excluded immune and endothelial cells [14]. This paracrine modality is not unique to TNBC and has been reported in other tissues including pancreas and prostate tumor, in which activation of Hh signaling in the tumor-adjacent mesenchyme—not in the epithelium—leads to accelerated tumor growth [20,21,22].

Despite the importance of the tumor-adjacent stroma in the oncogenicity of Hh signaling, no in vitro models have been developed that support robust Hh signaling and associated tumorigenicity. Animal studies have provided valuable information confirming the interaction between the tumor–stroma and disease progression [19,23,24], but are not amenable to precise monitoring and spatial manipulation of tumor–stroma interactions. Moreover, murine models of Hh signaling may not fully recapitulate the disease in patients; further strengthening the need for a human-based model for the preclinical evaluation of drug-sensitivity in a fast manner. The absence of in vitro models limits multi-endpoint assessment of therapeutic targets and identification of tumor–stromal interactions driving the oncogenicity of the Hh pathway in TNBC.

The studies performed seek to develop a paracrine model of Hh signaling using human breast-derived cells in which both the transcriptional activity in the tumor-adjacent stroma and tumorigenicity of the pathway are recapitulated to assess the impact of tumor–stroma proximity and mesenchymal cell subtypes in the oncogenic activity of the Hh pathway. In this study, we further confirmed a lack of sensitivity to Hh inhibition in TNBC cells and a paracrine interplay between the tumor and stroma in both in vitro and in vivo models. We adapted a previously developed microscale co-culture platform [25,26] to develop a paracrine model of Hh signaling in TNBC and examined the impact of mixed and adjacent culture modalities in the tumorigenicity and transcriptional activity of the Hh pathway. The culture model was composed of TNBC human cell lines and a subset of mesenchymal cells consisting of human mammary fibroblasts (HMFs), CAFs, tumor cells that undergo epithelial–mesenchymal transition (EMT), and adipose mesenchymal stem cells (ADMSCs). Our studies provide a human model of paracrine Hh signaling and show that the paracrine modality of the Hh pathway is supported by fibroblasts and tumor cells that undergo EMT via both soluble secreted factors and cell–cell interactions.

## 2. Methods

### 2.1. Cell Culture

Triple-negative (estrogen (ER), progesterone (PR), human epidermal growth factor receptor 2 (HER2), (ER^−^PR^−^HER2^−^)) breast cancer cell lines, MDA-MB-231, MDA-MB-468, and Hs578t; Luminal A (ER^+^PR^+^HER2^−^) MCF-7 and T-47D cells; and non-tumorigenic triple-negative 184B5 and MCF10A cell lines were purchased from ATCC. MDA-MB-231 expressing Green fluorescent protein (GFP) (GFP-MDA-MB-231) cells were obtained from Dr. Suranganie Dharmawardhane’s lab and were described in [27]. NIH3T3 were purchased from Sigma-Aldrich (St. Louis, MO, USA). The immortalized human mammary fibroblasts, RMF-621-tert cells (HMFs), derived from the stromal vascular fraction of a reduction mammoplasty [28], were obtained from Dr. Lisa Arndt’s lab (University of Wisconsin-Madison, WI, USA). Breast CAFs were gifted to us from Dr. Andreas Friedl’s lab (University of Wisconsin-Madison, WI, USA). MDA-MB-468, MDA-MB-231, MCF-7, HMFs, and NIH3T3 were cultured in Dulbecco’s Modified Eagle’s Medium (DMEM) high-glucose media with L-Glutamine (D5796, Sigma-Aldrich, St. Louis, MO, USA) supplemented with 10% heat-inactivated fetal bovine serum (FBS) (F6765, Sigma-Aldrich, St. Louis, MO, USA), and 1% penicillin/streptomycin (P/S) (P4333, Sigma-Aldrich, St. Louis, MO, USA). Hs578t were cultured in DMEM high-glucose media with L-Glutamine supplemented with 10% heat-inactivated FBS, 0.01 mg/mL human insulin (I9278, Sigma-Aldrich, St. Louis, MO, USA), and 1% P/S. T-47D were cultured in Roswell Park Memorial Institute (RPMI) medium 1640 with L-Glutamine (11875-093, Thermo Fisher Scientific, Grand Island, NY, USA) supplemented with 10% heat-inactivated FBS, 0.01 mg/mL human insulin, and 1% P/S. 184B5 were cultured in Mammary Epithelial Cell Growth Media (MEGM) culture medium Kit (CC-3150, Lonza, Walkersville, MD, USA) and supplemented with 1 ng/mL cholera toxin (C8052, Sigma-Aldrich, St. Louis, MO, USA) as recommended by ATCC. MCF10A cells were cultured in DMEM/Ham’s F-12 (11330032, Thermo Fisher Scientific, Grand Island, NY, USA) and supplemented with 0.01 mg/mL insulin, 500 ng/mL hydrocortisone, and 10% FBS. Adipose mesenchymal stem cells (ADMSC) were purchased from Lonza and sustained and supplemented by ADMSC Growth Medium Bullet kit (PT-4505, Lonza, Walkersville, MD, USA). All cells were mycoplasma-free and maintained at 37 °C in a 5% CO_2_ incubator. Passages were performed at 75%–80% confluence using 0.5% trypsin (59418C, Sigma-Aldrich, St. Louis, MO, USA). Primary cells and cell lines were used within 8 and 25 passages total, respectively. Viable cells were counted using CBA Vision Image Cytometer (Nexcelom Bioscience LLC, Lawrence, MA, USA) using the Trypan Blue (T8154, Sigma-Aldrich, St. Louis, MO, USA) exclusion method.

### 2.2. Co-Culture and Hh Drug Inhibition Studies

Fabrication of polystyrene-based microwells has been described in detail in several publications [25,26]. Tumor cells (5000 cells/microwell) and mesenchymal cell subtypes were seeded in adjacent compartments simultaneously. A total of 2000 cells (ADMSC) or 4000 fibroblasts were seeded per microwell to achieve confluence. Cells were incubated overnight for attachment before overlaying to initiate co-culture. For co-cultures with TGFβ-Hs578t, Hs578t was pre-treated with 10 µg/mL of transforming growth factor beta (TGF-β) (100-21, PeproTech, Rocky Hill, NJ, USA) for 72 h (replenished at 48 h) before seeding tumor cells. EMT in Hs578t was confirmed by immunostaining. Culture compartments were washed three times with phosphate-buffered saline (PBS) 1× (D8537, Sigma-Aldrich, St. Louis, MO, USA) to remove any remaining Transforming growth factor beta (TGF-β). A volume of 75 uL of low-serum culture medium (DMEM + 0.5% FBS + 1% P/S) containing PBS 1× (Vehicle) or 5 nM SHH (100-45, PeproTech, Rocky Hill, NJ, USA) +/− pharmacological Hh inhibitors was overlaid to interconnect the compartments for co-culture. The following concentrations of Hh inhibitors were used and selected based on suppression of the pathway: 5 µM NVP-LDE225 (NVP) (S2151, Selleckchem, Houston, TX, USA), 5 µM GANT61 (S8075, Selleckchem, Houston, TX, USA), and 3 µM cyclopamine (S1146, Selleckchem, Houston, TX, USA). A low serum medium is required for strong SHH signaling in the stroma as shown in a previous publication [29]. For co-culture with non-tumorigenic cell lines (184B5 and MCF10A) their basal culture media was used at a 0.5% FBS serum concentration. Half of the total cell culture volume was replaced after the first 48 h of co-culture for metabolic waste removal and nutrient/SHH/Hh inhibitors replenishment. Tumor cell growth was monitored at 96 h of co-culture using the Click-iT EdU Alexa fluor 594 imaging kit (C10339, Thermo Fisher Scientific, Grand Island, NY, USA) according to the manufacturer’s recommendations. Following 5-ethynyl-2’-deoxyuridine (EdU) incubation, the compartment containing MDA-MB-468 cells was imaged for cell proliferation analysis using the ZOE Fluorescent Cell Imager and an Olympus IX71 fluorescence microscope at a magnification of 20× and 4×, respectively. Nuclear and EdU stains were quantified by ImageJ to obtain total and EdU + tumor cell counts.

### 2.3. Gene Expression Analysis

The gene expression level was analyzed using RT-qPCR. For in vitro cell culture, after 48 h of SHH treatment, cells were lysed independently in each compartment as facilitated by the use of adjacent culture regions and open device design. mRNA was isolated directly from cell lysates using Dynabeads mRNA DIRECT Micro Kit (61021, Invitrogen, Carlsbad, CA, USA). Meanwhile, tumors from the xenograft mice model were preserved in RNAlater (R0901, Sigma-Aldrich, St. Louis, MO, USA) and stored in −20 °C for genomic analysis. Approximately 10 mg of the tumors were cut with a blade and homogenized with a conventional rotor. Total RNA was collected and purified using the RNeasy Mini Kit Part 1 and Part 2 for on-column DNase digestion (Qiagen, GermanTown, MD, USA). The total RNA concentration was quantified using a Nanodrop spectrophotometer. For both, in vitro and in vivo experiments, total mRNA (20–30 ng) was reverse transcribed to generate cDNA and quantified using TaqMan™ RNA-to-CTTM 1-Step kit (4392938, ThermoFisher Scientific, Grand Island, NY, USA) with the StepOne Real-time PCR System (Thermo Fisher Scientific, Grand Island, NY, USA). Mouse and Human GLI1 (Mm00494654_m1 and Hs00171790_m1), PTCH1 (Mm00436026_m1 and Hs00970976_m1), and SMO (Hs01090242_m1) TaqMan™ primers (Thermo Fisher Scientific, Grand Island, NY, USA) were used to determine the relative expression of Hh genes. Expression levels were quantified relative to Glyceraldehyde-3-Phosphate Dehydrogenase (GAPDH) (Mm99999915_g1 and Hs02758991_g1) (Thermo Fisher Scientific, Grand Island, NY, USA).

### 2.4. Tumor Xenograft Studies

As MDA-MB-468 cells secrete small amounts of SHH-ligand in vitro, tumor cells were co-injected with beads saturated with the active form of the SHH-ligand. Affi-Gel Blue beads have been used in vivo for sustained diffusion of SHH-ligand [30,31]. The preparation of Affi-Gel Blue beads (1537302; Bio-Rad Laboratories, Hercules, CA, USA) consisted in the incubation with 400 ug/mL of human recombinant SHH-ligand (78065, STEMCELL Technologies, Inc., Vancouver, Canada) with 0.1% bovine serum albumin (BSA) (A7906, Sigma Aldrich, St. Louis, MO, USA) carrier protein (SHH+) or 0.1% BSA (Vehicle) for ≥1 h at 37 °C. Affi-Gel Blue beads were rinsed 4 times in PBS 1× and manually selected for uniform size. Protein-soaked beads were stored at 4 °C for a maximum of 1 week. Severe combined immunodeficient (SCID) Hairless Outbred (SHO) female mice that were 3 weeks old (Charles River Laboratories, Wilmington, MA, USA) were segregated into eight groups (*n* = 9 mice/group). Half of the groups were injected with MDA-MB-468 (1 × 10^6^) alone, while the other half were injected with MDA-MB-468 (1 × 10^6^) + ADMSC (2.5 × 10^5^). Cells were mixed with 1:1 Matrigel (CB40230A, Fisher Scientific, Pittsburgh, PA, USA) in starvation media [32,33] and co-injected with an average of 100 beads in the mammary fat pad of mice. NVP drug was dissolved in dimethyl sulfoxide (DMSO) (D2650, Sigma-Aldrich, St. Louis, MO, USA) and corn oil (1.5%) (sc-214761, Santa Cruz Biotechnology, Dallas, TX, USA) and then diluted in the carrier 0.5% sodium carboxymethyl cellulose (419273-100G, Sigma-Aldrich, St. Louis, MO, USA). After 2 weeks post-injection, mice were orally gavaged daily with Vehicle or 20 mg/kg/day NVP-LDE225 for 4 weeks. Tumor formation was measured with calipers and monitored weekly for 6 weeks. Tumor volumes were calculated as the volume of an ellipsoid using the formula: V = (π/6) × L × W × H as in [32,33]. Animal experiments were reviewed by the Institutional Animal Care and Use Committee at Universidad Central del Caribe (UCC) at Bayamón and approved under protocol number #051-2017-08-IBC-PHA on 11th April 2016.

### 2.5. Patient Sample Analysis

The RNA-samples used were derived from de-identified breast tumor tissues and studies were approved by the Ponce Health Science University IRB Committee under project number 160212-PC on 3rd March 2016. Expression levels of Hh target genes were evaluated in a total of 20 tumors and 10 paired “normal-adjacent tissue” from fresh-frozen tumor samples from Hispanic breast cancer patients from Puerto Rico (PR). The genomic material was provided for analysis through a collaboration with the PR BioBank. Patient consent was obtained for all samples by the PR Biobank at Ponce Health Sciences University. Receptor status and PanCancer subtype were confirmed by a pathologist and 150 µg of total RNA per sample were evaluated using the PanCancer Pathways Panel (Nanostring Technologies, Inc, Seattle, WA, USA) in all tumor samples. Tumor xenografts collected at 2 weeks post-inoculation were used to monitor Hh signaling and other pathways in response to the active form of SHH-ligand. Differentially expressed genes (DEGs), gene set analysis (GSA), and pathway scoring were performed using nCounter (R) Advanced Analysis Plugin for nSolverTM software. DEGs are extracted by modeling the log2 expression of each gene in response to multiple conditions using a linear regression approach. Since multiple hypothesis tests are performed to state the statistical significance of each gene, the p-values are corrected using the Benjamini–Yekutieli (BY) method to control the false discovery rate. GSA calculates global significance scores for each gene in a particular pathway and KEGG annotation is used to generate these gene sets. Finally, pathway or deregulation scores are generated using principal component analysis once genes are mapped to particular pathways and their expression is scaled across samples. Adjusted ** *p*-value < 0.005.

### 2.6. Adjacent versus Mixed Culture Studies

The microscale open co-culture device design consists of two microwells (9 mm^2^) within a larger parent well (28.3 mm^2^). For the macro-scale device, the culture region of microwells was increased by a factor of 9 as compared to the microscale device. The volume-to-cell ratio was kept constant at 0.01 µL/cell for both macro and microscale devices. NIH3T3 were seeded forty-eight (48) hours before beginning co-culture to ensure growth arrest and a uniform monolayer. For the CAF-like phenotype, NIH3T3 were pre-treated with 0.8 nM TGF-β in culture flasks for 72 h (replenished at the 48 h) before cell seeding in microwells. For microscale, 5000 MDA-MB-231 GFP^+^ cells and 2500 fibroblasts (NIH3T3 or TGF-β-NIH-3T3) were seeded per device, either in the same compartment (mixed) or adjacent compartments. For macroscale, 45,000 and 22,500 of MDA-MB-231 GFP^+^ and fibroblasts were seeded, respectively, per device. Cultures were treated with 5 µM NVP-LDE225 (S2151, Selleckchem, Houston, TX, USA) diluted in DMEM HG supplemented with 0.5 g/L L-glutamine, 0.5% FBS, and 1% (P/S). Adjacent cultures were supplemented with 5 nM SHH (100-45, PeproTech, Rocky Hill, NJ, USA) to ensure pathway activation. Cells were co-cultured for 96 h. The tumor cell growth was quantified based on total fluorescent cell counts relative to the tumor monoculture at 96 h using the CBA Vision Image Cytometer (Nexcelom Bioscience LLC, Lawrence, MA, USA).

### 2.7. ALDH1 Detection Assay

Expression of ALDH1 was examined after 96 h of culture. Each well was washed once with PBS 1× and incubated for seven minutes at 5% CO_2_ 37 °C with Accutase 1× solution (A6964, Sigma-Aldrich, St. Louis, MO, USA) for cell detachment. Cells were resuspended in warm cell culture media (DMEM HG + L-glutamine + 10% FBS + 1% P/S) and adjusted to 10^5^ cells per sample. ALDH1^+^ expression was detected in cell samples using the AldeRed ALDH Detection Assay (SCR150, Millipore, St. Louis, MO, USA) according to the manufacturer’s recommendation. Briefly, cells were centrifuged and resuspended in 100 µL of AldeRed assay buffer supplemented with Verapamil and AldeRed reagent. Diethylaminobenzaldehyde reagent, an ALDH1 inhibitor was used in some samples as a negative control to identify the ALDH1-positive (ALDH1^+^) population. Samples were analyzed by performing a dual fluorescence assay measuring the intensity of green and red fluorescence using a CBA Vision Image Cytometer.

### 2.8. Immunofluorescent Staining

The staining process was carried out at room temperature (25 °C). Cells were fixed with 4% paraformaldehyde for 20 to 30 min. Cell permeabilization was performed using 0.5% Triton (T8787, Sigma-Aldrich, St. Louis, MO, USA) for 10 min. Devices were washed twice with PBS 1× and placed on an orbital shaker with 3% BSA + 0.1% Tween 20 (P9416, Sigma-Aldrich, St. Louis, MO, USA) ) in PBS 1× solution at 300 rpm for 1 h. Cells were incubated with the following primary antibodies: anti-alpha smooth muscle actin (ab7817, Abcam, Cambridge, MA, USA), anti-alpha tubulin (acetyl K40) (ab24610, Abcam, Cambridge, MA, USA), anti-fibroblast activation protein (ab53066, Abcam, Cambridge, MA, USA), anti-SHH (ab53281, Abcam, Cambridge, MA, USA), and anti-vimentin (ab92547, Abcam, Cambridge, MA, USA) at a ratio of 1:200, 250, 250, 250, and 250, respectively, in 3% BSA + 0.1% Tween 20 in PBS 1× solution. Devices were placed on an orbital shaker at 300 rpm for 1 h and then washed three times with 0.1% Tween 20 in PBS 1× solution, waiting 10 min between each wash. Cells were then incubated with the following secondary antibodies: Alexa 488 (ab150117, Abcam, Cambridge, MA, USA) and Alexa 594 (ab150084, Abcam, Cambridge, MA, USA) at a ratio of 1:500 in 3% BSA + 0.1% tween 20 in PBS 1× solution for 1 h. Devices were placed on an orbital shaker and washed three times with PBS 1× prior to staining with Hoechst 33342 nuclear dye (1:500) for 30 min. Fluorescent images were obtained with an Olympus IX71 laser scanning confocal microscope using 10×, 20×, 40×, and 60× magnification objectives.

### 2.9. Immunohistochemistry (IHC)

Tissues from tumor xenografts in mice were fixed in buffered formalin (HT501128, Sigma-Aldrich, St. Louis, MO, USA) and transported to an independent private laboratory (Southern Pathology, Ponce, PR, USA) for IHC and tissue pathology. Briefly, tissues were formalin-paraffin embedded, sliced, and stained with hematoxylin/eosin (H&E), Ki67 (Ki67-MIB-1, Dako Omnis-Agilent Technologies, Carpinteria, CA) and SMA (SMA-14A, Dako Omnis-Agilent Technologies, Carpinteria, CA) primary antibodies, followed by incubation with HRP secondary antibodies. SMA protein stain was used to confirm the infiltration of the mouse stroma in each tumor biospecimen. Two tumors were examined per each experimental condition. Tumor tissue sections were imaged at 50× magnification using a Dako Omnis instrument. An experienced tumor pathologist, who was blinded to the experimental treatment groups, scored each tissue staining.

### 2.10. Western Blot

Cells were lysed using sample buffer (62.5 mM Tris-Cl, pH 6.8, 10% glycerol, 2% SDS, 5% b-mercaptoethanol) and bromophenol blue to yield whole-cell extracts. Whole-cell extracts were boiled, and protein concentration was determined using an RC DC Protein Assay kit (5000121, Bio-Rad Laboratories, Inc., Richmond, CA, USA) following the manufacturer’s instructions and were measured on a Genesys 5 spectrophotometer (Spectronic Instruments, Inc., Fitchburg, WI USA). Samples were resolved in a 10% sodium dodecyl sulfate polyacrylamide gel electrophoresis (SDS-PAGE) gel. Proteins were transferred to a 0.45 µm PVDF membrane (10600023, Sigma-Aldrich, St. Louis, MO, USA) in a tris-glycine transfer buffer with 20% methanol using a Trans-Blot Cell (Bio-Rad Laboratories, Inc., Richmond, CA, USA). The membranes were pre-blocked in a solution of 5% dry-cow milk, 0.02% sodium azide, and 0.2% Tween 20 in PBS 1× (PBST). Membranes were then incubated overnight in the same solution containing primary antibody for anti-SHH (ab53281, Abcam, Cambridge, MA, USA), anti-PTCH1 AV44249 (Sigma-Aldrich, St. Louis, MO, USA), and anti-Su (fu) (sc-137014, Santa Cruz Biotechnology, Dallas, TX, USA). Blots were washed with PBST solution before incubation with secondary antibodies conjugated to horseradish peroxidase (ab6789 and ab6721, Abcam, Cambridge, MA, USA) diluted in the identical solution without sodium azide for 2 h. Expression levels of beta-actin (A5441, Sigma-Aldrich, St. Louis, MO, USA) were used as a loading control to ensure equivalent loading of samples. Protein bands were visualized by enhanced chemiluminescence (sc-2048, Santa Cruz Biotechnology, Dallas, TX, USA). Band intensities were quantified by optical density analysis (LabWorks 4.0 Image Acquisition and Analysis Software; UVP BioImaging Systems, UVP Inc., Upland, CA, USA). Full western blots can be found in Supplementary Figures S1 and S2.

### 2.11. Statistical Analysis

For animal studies, evaluation of normality precept was done using the Shapiro–Wilk estimator. Presence of outliers was verified via Grubbs’ test. Bivariate analyses consist of ordinary one-way ANOVA and paired samples *t*-test to detect mean changes between treatments and cell combinations. To evaluate mean changes across time, a general linear model repeated-measures ANOVA approach was used. Mauchly’s test of sphericity was used to assess compound symmetry in our model; if non-significant (*p* > 0.05), we report the Greenhouse–Geisser epsilon correction; if significant (*p* < 0.05), Pillai’s trace estimator was reported. Dunnett’s adjustment was used to perceive statistical differences between and within the groups via experimental concentration as a fixed factor. The significance level (α) was set to ≤0.05, except for the normality diagnostic test (*p* > 0.05). IBM SPSS, (Chicago, IL, USA) V.23.0 for Windows and GraphPad Prism 7 (GraphPad Software, San Diego, CA, USA) were used. For in vitro studies, multifactorial analysis using one-way and two-way ANOVA was performed to detect significant changes. Two-sample *t*-tests were done to compare changes concerning baseline values or treatment controls. Wilcoxon–Mann–Whitney tests were performed only if the data did not follow a normal distribution. *p*-values less than 0.05 were considered significant as follows: * *p* < 0.05, ** *p* < 0.01, *** *p* < 0.001, **** *p* < 0.0001.

## 3. Results

### 3.1. Hh Inhibitors and SHH-Ligand Had Limited Growth Effects in Tumor Monocultures

**Figure 1:**
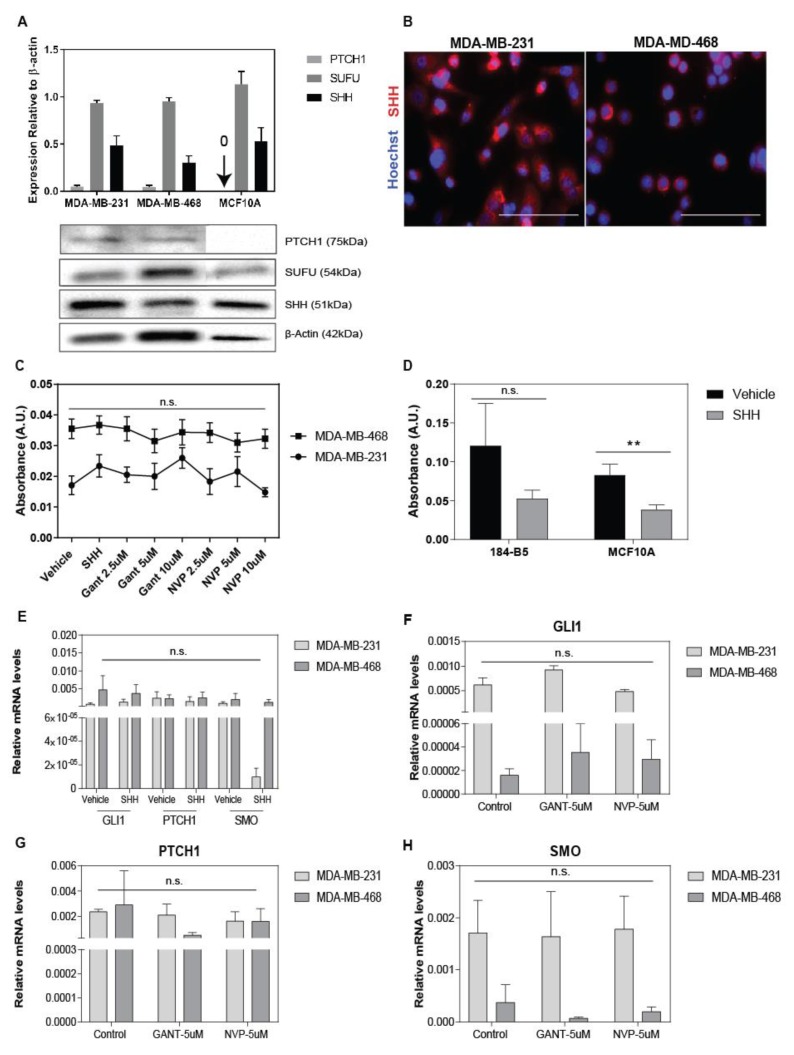
Hedgehog (Hh) inhibitors do not suppress canonical Hh signaling and growth of triple negative breast cancer (TNBC) cells. (**A**) Expression of Sonic hedgehog (SHH)-ligand (51 kDa), patched1 (PTCH1) receptor (75 kDa), and suppressor of fused homolog (SUFU) (54 kDa) in tumor cells relative to β-actin (42 kDa). Data represents the relative mean intensity +/− standard error of mean (SEM) of three to four independent experiments. Representative protein bands are shown in the image below the bar graph. (**B**) Immunofluorescent staining of SHH expression in MDA-MB-231 and MDA-MB-468 cells. SHH (red) and Hoechst (blue). Scale bar = 100 µm. (**C**) Tumor cell viability was evaluated in response to 5 nM SHH-ligand and 2.5, 5, and 10 µM concentrations of NVP-LDE225 (NVP) and GANT61 (GANT) for 96 h using the XTT assay. Data represent mean +/− SEM of three independent experiments with *n* = 4. (**D**) Viability of non-tumorigenic breast cell lines treated with 5 nM SHH-ligand at 96 h. Data represent mean +/− SEM of three independent experiments with *n* = 4. Significance determined by Student *t*-test ** *p*-value < 0.01. (**E**–**H**) Expression levels of glioma associated oncogene family zinc finger 1 (*GLI1*), *PTCH1*, and Smoothened (*SMO*) genes relative to Glyceraldehyde-3-Phosphate Dehydrogenase (*GAPDH*) exposed to exogenous SHH-ligand for 24 h (**E**) and Hh inhibitors, GANT (5 µM) and NVP (5 µM) for 48 h (**F**–**H**). Data represent mean +/− SEM of three to four experiments with *n* = 3–4.

To determine the sensitivity of breast tumor cells to Hh inhibitors, expression levels of Hh pathway components and cell growth were evaluated in response to exogenous addition of SHH-ligand, SMO, and GLI1 inhibitors, NVP-LDE225 (NVP) and GANT61, respectively. Expression of the full-length of SHH-ligand (51 kDa), SUFU (54 kDa), and PTCH1 receptor (75 kDa) were confirmed in all breast cell lines except for SHH-ligand in MCF-7 cells (Figure 1A,B, Figure S3A,B). MDA-MB-231 expressed the highest endogenous levels of SHH-ligand as compared to MDA-MB-468, MCF-7, and T-47D. In contrast to previous research [34], where changes in *PTCH1*, *GLI1*, and *SMO* transcripts were detected in TNBC cell lines, we did not observe an increase in these transcripts in response to SHH-ligand treatment (Figure 1C,E). Similarly, an increase in the levels of these transcripts was not detected in 184B5, MCF-7, and T-47D cells in response to SHH-ligand treatment (Figure S3D. Evaluation of pharmacological Hh inhibitors at increasing concentrations within the micromolar range did not affect the viability of TNBC cells (Figure 1C). The expression levels of *PTCH1*, *SMO*, and *GLI1* transcripts were not affected by GANT61 and NVP treatments, thus confirming the absence of Hh signaling suppression in TNBC cells (Figure 1F–H). Contrary to TNBC cells, significant changes in cell viability were detected in non-tumorigenic breast cells and breast cancer cells of the luminal A subtype. Non-tumorigenic breast cell lines, 184B5 and MCF10A, showed reduced cell viability in response to the exogenous addition of SHH-ligand (Figure 1D). T-47D cells showed a significant decrease in cell viability when treated at concentrations above 2.5 µM for both GANT61 and NVP. An opposite behavior to T47D was observed in MCF-7 cells in which increasing concentrations of NVP stimulated cell growth (Figure S3C). Changes in cell viability in both cell lines did not correlate with suppression of *GLI1*, *PTCH1*, or *SMO* levels (Figure S3E–G). Henceforth, changes observed in the behavior of Luminal A cell lines treated with Hh inhibitors did not correlate with suppression of *GLI1*, *PTCH1*, and SMO transcripts, thus changes observed in tumor cell behavior are likely the result of off-target effects or non-canonical Hh signaling mechanisms [35].

### 3.2. Non-Tumorigenic Cells Are a Main Therapeutic Target of Hh Inhibitors in TNBC Tissues

**Figure 2:**
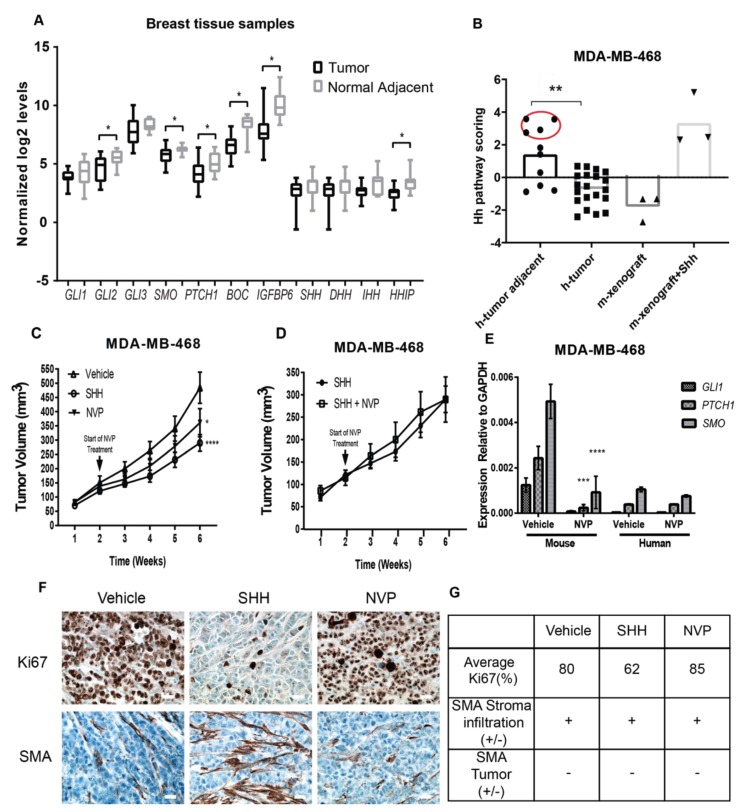
Hedgehog (Hh) signaling in breast tumor tissues and a mouse xenograft model of triple negative breast cancer (TNBC). (**A**) Hh pathway scores for a total of 20 tumors and 10 paired “normal-adjacent tissue” from fresh tumor samples from breast cancer patients in Puerto Rico (PR) were evaluated using the PanCancer pathways array from Nanostring. MDA-MB-468 xenograft tumors +/− Sonic hedgehog (SHH)-ligand collected at two weeks post-injection were used as negative and positive controls for SHH-signaling. TNBC samples are highlighted in red. Adjusted ** *p*-value < 0.005. (**B**) The normalized log2 expression for Hedgehog-pathway genes across tumor and normal adjacent breast tissue samples are shown in (**A**). Significance determined by Student *t*-test * *p*-value < 0.05. (**C**,**D**) Tumor growth curves in xenograft tumors composed by MDA-MB-468 +/− SHH-ligand. NVP or Vehicle was administered daily during the last four weeks. Data shows mean +/− standard error of mean (SEM) of nine mice per group. Significance was determined via ANOVA analysis. * *p*-value < 0.05, **** *p*-value < 0.0001. (**E**) Expression levels of glioma associated oncogene family zinc finger 1 (*GLI1*) and patched1 (*PTCH1*) transcripts relative to Glyceraldehyde-3-Phosphate Dehydrogenase (GAPDH) in xenografts tumors and NIH3T3 cells using human and mouse-specific primers. Data shows mean +/− SEM of six mice per condition. Significance was determined via ANOVA analysis, Adjusted *** *p*-value < 0.001 and **** *p*-value < 0.0001. (**F**) Representative immunohistochemistry staining from mouse tumor xenografts treated with Vehicle, NVP, and SHH-ligand. Scale bar = 20 µm. (**G**) Average values of Ki67 and SMA staining for two tumors of each experimental treatment were summarized based on the reported values in the pathology report.

To determine whether Hh signaling activity is present in the tumor-adjacent tissue of breast cancer patients, the expression levels of Hh transcripts were evaluated in a panel of 20 tumors and 10 paired “normal-adjacent tissue” specimens from breast cancer patients. Scoring of Hh pathway genes collectively shows elevated levels of Hh target genes in the tumor-adjacent stroma but not in the bulk tumor tissue, particularly of the TNBC subtype (Figure 2A). Examination of main Hh target genes individually shows higher levels of *GLI2*, *PTCH1*, *SMO*, *BOC*, and *HHIP* transcripts in the tumor-adjacent tissue as compared to the bulk tumor (Figure 2B), further supporting the importance of the tumor-adjacent tissue in the activity of Hh signaling.

To confirm that NVP (also known as Erismodegib and Sonidegib) is suppressing canonical Hh signaling in non-tumorigenic cells, tumor volume and Hh target genes were examined in a mouse xenograft model composed of Matrigel-embedded MDA-MB-468 cells. Hh transcripts of human and murine specificity were used to distinguish MDA-MB-468 from cells of mouse tissue origin. Tumor volume significantly decreased at four weeks post-treatment with both NVP and SHH-ligand (Figure 2C). NVP had no added benefit when combined with SHH-ligand (Figure 2D). Examination of Hh transcripts indicate that tumor growth suppression correlates with down-regulation of *GLI1*, *PTCH1*, and *SMO* transcripts in cells of mouse but not of human origin (Figure 2E). In agreement with the reduced tumor growth rates observed (Figure 2C), the immunohistochemical analysis of Ki67, a proliferation marker and prognosis factor used in the examination of the pathology of tumors, indicate a 20% reduction in the Ki67 levels in tumors treated with SHH-ligand (Figure 2F–G). The levels of Ki67 were not affected by NVP treatment. SMA staining was positive in non-tumor cells, confirming the tumor infiltration of the mouse stroma (Figure 2F–G).

This data confirms a lack of suppression of the canonical modality of Hh signaling in MDA-MB-468 cells treated with NVP, and supports that other non-tumorigenic cells are targets of the therapeutic response to Hh inhibitors.

### 3.3. Fibroblasts are Therapeutic Targets of Hh Inhibitors

Fibroblasts are the main component of the adjacent tumor stroma [36] and several studies support their role in paracrine Hh signaling [37,38]. However, whether this oncogenic role is unique to CAFs or involves other fibroblasts as well is unknown, as fibroblast subtypes are indistinguishable in tissues based on the examination of main pathological markers such as alpha-smooth muscle actin (SMA), vimentin (VIM), and fibroblast-activated protein (FAP), yet they are important for proper modeling of this interaction in vitro. In this study, Hh signaling activity and tumorigenicity were examined in fibroblasts from diverse sources. The NIH3T3 cell line and primary breast fibroblasts (HMFs and CAFs) were selected for evaluation. NIH3T3 fibroblasts were used as a positive control because it is a well-known established cell line that consistently gives a robust signal in response to SHH-ligand [39]. NIH3T3 were treated with TGF-β as a positive control of a CAF-like phenotype. Expression of the main fibroblast markers SMA, VIM, and FAP was confirmed in both NIH3T3 and primary fibroblasts (Figure S4A). PTCH1 receptor was expressed in HMFs and NIH3T3, a very weak protein band was observed in CAFs (Figure S4B,C). A full-length SHH-ligand was found in all fibroblasts, but very low levels were observed in NIH3T3 cells (Figure S4B). Primary cilium and SUFU was found in all fibroblasts examined (Figure S5). As expected, *GLI1* levels were overexpressed in response to SHH-ligand in NIH3T3, confirming Hh signaling activity (Supplementary Figure S4C). HMFs had increased levels of *SMO* and *GLI1* in response to exogenous SHH-ligand but were only significant for *SMO* (Figure S4C). SMO is a positive regulator of the pathway and will counteract the negative feedback loop driven by the PTCH1 receptor in the canonical response to SHH-ligand. The increase in *SMO* levels observed in HMFs should be investigated further as these may lead to significant growth regulatory mechanisms associated with the pathology and hyperactivity of the pathway in adult breast tissues through SMO-dependent non-canonical [40] and canonical Hh signaling mechanisms [17].

**Figure 3:**
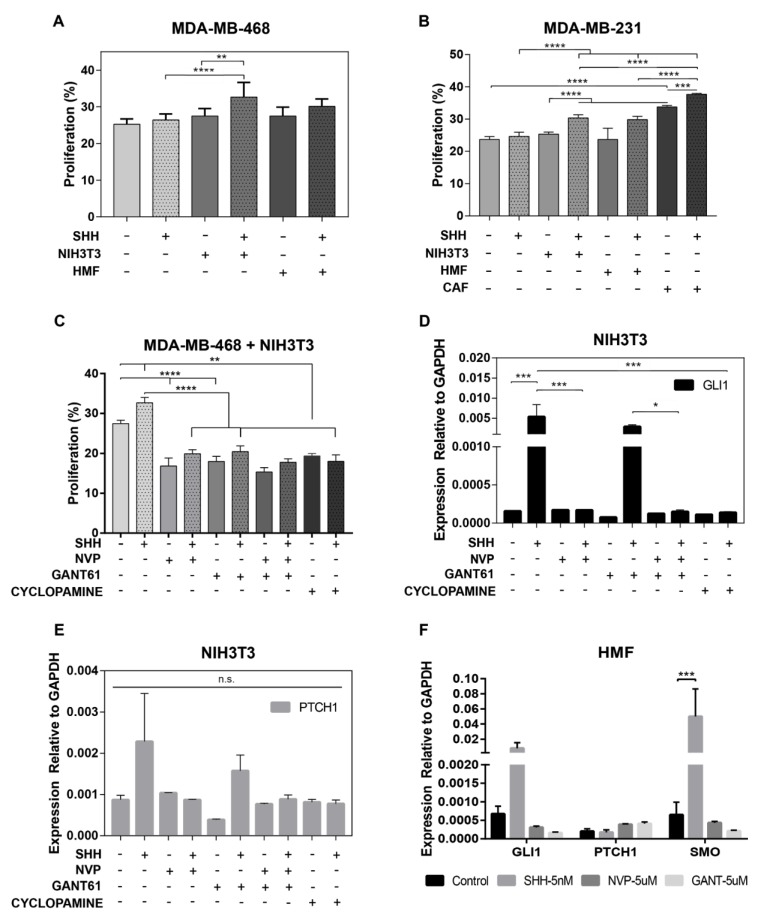
Fibroblasts are a therapeutic target of Hedgehog (Hh) inhibitors in triple negative breast cancer (TNBC). (**A**) Proliferation of MDA-MB-468 in presence and absence of Sonic hedgehog (SHH)-ligand (5 nM) in mono and co-culture with NIH3T3 and human mammary fibroblasts (HMF) cells during 96 h. Data represents mean +/− SEM of one to four experiments with *n* = 7–12. (**B**) Proliferation of MDA-MB-231 in presence and absence of SHH-ligand (5 nM) in mono and co-culture with NIH3T3, CAF, and HMF cells during 96 h. Data represents mean +/− standard error of mean (SEM) of one to four experiments with *n* = 7–14. (**C**) Proliferation of MDA-MB-468 in co-culture with NIH3T3 and HMFs treated with 5 nM SHH-ligand and Hh inhibitors, NVP, GANT61, and cyclopamine for 96 h. Data represents mean +/− SEM of two to three experiments with *n* = 3–8. (**D**,**E**) Expression levels of glioma associated oncogene family zinc finger 1 (*GLI1*) and patched1 (*PTCH1*) relative to Glyceraldehyde-3-Phosphate Dehydrogenase (*GAPDH*) in NIH3T3 cells treated with 5 nM SHH-ligand and Hh inhibitors for 24 h. Data represent mean +/− SEM of three experiments with *n* = 2–3. (**F**) Expression levels of *GLI1*, *PTCH1*, and Smoothened (*SMO*) relative to *GAPDH* in HMF cells treated with 5 nM SHH-ligand and Hh inhibitors for 24 h. Data represent mean +/− SEM of three experiments with n = 2–4. Significance was determined via ANOVA analysis * *p*-value < 0.05, ** *p*-value < 0.01, *** *p*-value < 0.001, **** *p*-value < 0.0001.

The proliferation of MDA-MB-231 and MDA-MB-468 cells was evaluated in tumor-adjacent fibroblast cultures treated with SHH-ligand and pharmacological Hh inhibitors (NVP, cyclopamine, and GANT61) to examine the tumorigenic effect of the selected fibroblasts. A custom open-multi-culture platform was used for co-culture to maintain a 1:1 tumor and stromal ratio, while keeping local confluence of the stroma needed for growth arrest and strong signal response to SHH-ligand [41]. The microwell platform consisted of adjacent microwells contained within a parent well, as described before [25]. Results show that the tumor cell growth was driven in response to active Hh signaling in all fibroblasts examined (Figure 3A,B). Tumor growth stimuli were stronger in MDA-MB-231 cells co-cultured with NIH3T3 or CAFs (Figure 3B). *GLI1* levels were upregulated in response to exogenous SHH-ligand in both HMFs and NIH3T3 (Figure 3D,F). *PTCH1* levels were significantly upregulated in NIH3T3 but not HMFs. Tumor growth was suppressed by treatment with Hh inhibitors (Figure 3C), and this inhibitory effect correlated with the downregulation of *GLI1* and *PTCH1* levels in fibroblasts (Figure 3D–F). Tumor cell growth was not significantly enhanced in T-47D and MCF-7 co-cultured with HMFs (Figure S6). Overall, the data here confirms fibroblasts as targets of Hh inhibitors in TNBC.

### 3.4. Impact of Mixed vs Adjacent Culture Modalities

**Figure 4:**
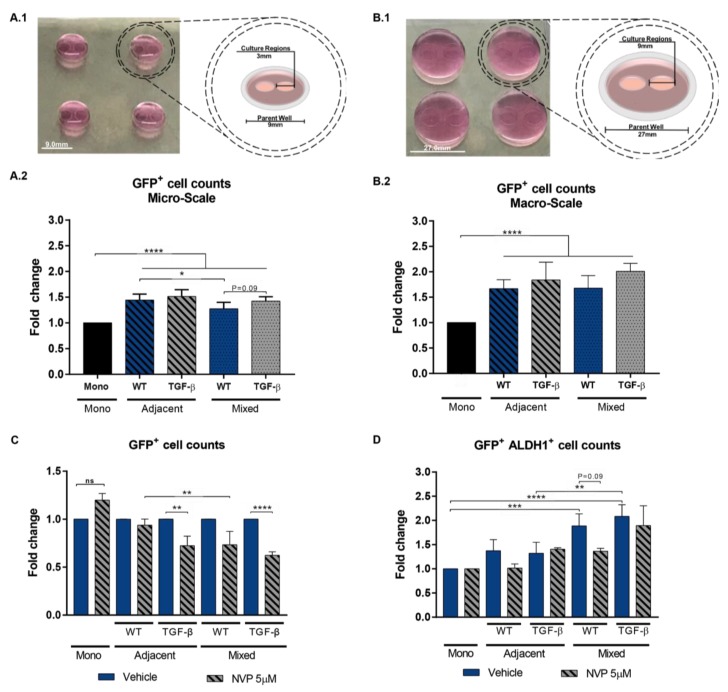
Impact of fibroblast subtypes and proximity among co-cultures. (**A.1**,**B.1**) Image and schematic of microscale and macroscale microwell culture platforms used for co-culture studies. (**A.2**,**B.2**) MDA-MB-231 Green fluorescent protein (GFP)^+^ were cultured alone or in mixed or adjacent cultured modalities with wild-type (WT) or transforming growth factor beta (TGF-β) pre-treated NIH3T3 fibroblasts. Tumor cell growth was quantified by counting the total tumor cells based by selecting for GFP^+^ expression relative to the tumor cell monoculture. (**C**) Cultures were treated with NVP for 96 h. Data shows the average mean of total GFP^+^ cell counts relative to Vehicle. (**D**) ALDH1 expression was quantified in tumor cells after 96 h in culture using the AldeRed ALDH detection assay. Dual expression quantification method was used to obtain the fraction of GFP^+^ALDH1^+^ cells. Data is presented with the mean +/− SEM of three independent experiments with *n* = 3–6. The data was analyzed using Student’s *t*-test. * *p*-value < 0.05, ** *p*-value < 0.01, *** *p*-value < 0.001, **** *p*-value < 0.0001.

Paracrine signaling plays an essential role in TNBC, where studies have prioritized this behavior primarily mediated by tumor-adjacent CAFs [24]. This paracrine interaction can be modeled via both adjacent and mixed tumor-CAF cultures, yet no studies have been done to determine the impact of culture modalities and fibroblast subtype in the observed tumor behavior. Here we explore the impact of both culture modalities, and fibroblast vs. CAF-like cells on the growth and expression of CSC markers in MDA-MB-231 cells. We used GFP-tagged (GFP) MDA-MB-231 for fast-tracking and identification of tumor cells, particularly in mixed cultures. NIH3T3 wild-type (WT) and NIH3T3 pre-treated with TGF-β were evaluated as models of fibroblasts and CAF-like cells. ALDH1, a well-established marker of breast cancer stem cells (CSCs) [24], was used to monitor changes in the CSCs proportion. The two-adjacent microwell culture device [25] (Figure 4A.1) was scaled-up by a factor of nine (Figure 4B.1) to obtain sufficient tumor cells for quantification of ALDH1 expression using image-based cytometry. Prior to tumor growth studies, Hh signaling activity was examined across culture modalities and compared to monocultures supplemented with SHH-ligand to determine whether GFP-tagged MDA-MB-231 secreted enough SHH-ligand to induce a robust Hh signaling activity in NIH3T3. Results indicate that *Gli1*, *Ptch1*, and *Smo* murine transcripts reached the highest levels in NIH3T3 monocultures treated with SHH-ligand, followed by a medium response in mixed co-cultures (Figure S7). Hh transcripts were low in adjacent co-cultures, suggesting that the diffusivity and local concentration of tumor-secreted SHH-ligand are limited, and indicating that supplementation with exogenous SHH-ligand is needed for robust Hh signaling in the tumor-adjacent NIH3T3 compartment. Tumor and fibroblasts cells were co-cultured for 96 h, and the NVP compound was used to suppress Hh signaling. Tumor growth was evaluated based on total cell counts in both microscale and macroscale devices to rule-out any platform-related artifact. Results show that tumor cell growth was significantly enhanced in both adjacent and mixed cultures as compared to the tumor monoculture condition (Figure 4A.2,B.2). This paracrine growth effect was similar across both macroscale and microscale culture platforms, indicating that the observed results are associated with tumor–fibroblast interactions and not culture platform artifacts. Although a slight increase of marginal significance in the total number of tumor GFP^+^ cells was detected for co-cultures with TGF-β treated NIH3T3 in the microscale platform (Figure 4A.2), the overall effect on tumor growth was of similar magnitude regardless of the culture modality for both WT and TGF-β co-cultures. Tumor growth was reduced in all co-cultures treated with NVP, but this effect was most potent for TGF-β cultures (Figure 4C). The proportion of GFP^+^ALDH1^+^ tumor cells was significantly enhanced in the mixed culture modality only for WT and TGF-β NIH3T3 co-cultures (Figure 4D). The population of ALDH1^+^ cells was suppressed only in WT co-cultures treated with NVP (Figure 4D), confirming the dependence on Hh pathway activity. This suppressive effect was not observed in co-cultures with TGF-β treated NIH3T3, which suggests that other non-Hh signaling mechanisms are supporting the increase in the ALDH1^+^ population.

### 3.5. Cells that Undergo EMT Support the Tumorigenicity of Paracrine Hh Signaling

**Figure 5:**
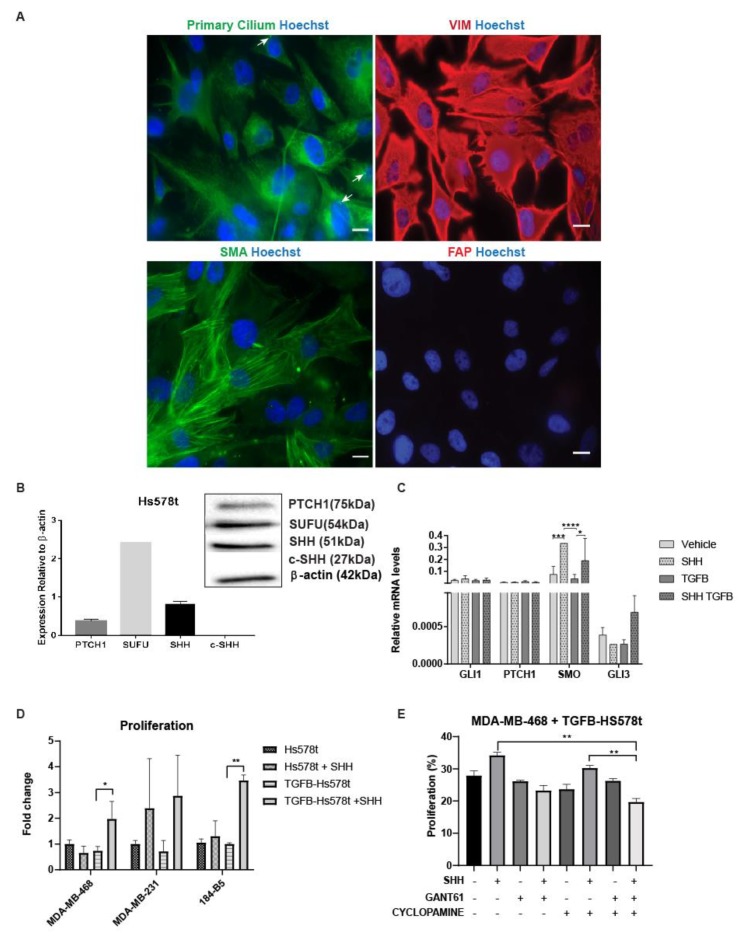
TGF-β-Hs578t supports paracrine Hedgehog (Hh) signaling. (**A**) Staining of mesenchymal markers acetylated-alpha tubulin (primary cilium), vimentin (VIM), alpha-smooth muscle actin (SMA), and fibroblast-activated protein (FAP). White arrows indicate sample primary cilium staining. The color blue in all the images represents nuclear staining using Hoechst. Images were digitally enhanced using Fiji for better visualization of mesenchymal markers. Scale bar = 10 µm. (**B**) Expression of patched1 (PTCH1) receptor (75 kDa), suppressor of fused homolog (SUFU) (54 kDa), and Sonic hedgehog (SHH)-ligand, full length (51 kDa) and c-product subunit (27 kDa) quantified relative to β-actin. Data represents the mean +/− standard error of mean (SEM) of four independent experiments, n = 2–3. Representative western blot bands were included. (**C**) Expression levels of Hh target genes glioma associated oncogene family zinc finger 1 (*GLI1*), patched1 (*PTCH1*), Smoothened (*SMO*), and *GLI3* in Hs578t treated with SHH-ligand and transforming growth factor (TGF)-β. Data represent mean +/− SEM of three to six independent experiments with n = 3. Significance was determined via ANOVA analysis * *p*-value < 0.05, *** *p*-value < 0.001, **** *p* < 0.0001. (**D**) Triple-negative breast cell lines were co-cultured adjacent to Hs578t or TGFβ-Hs578t for EMT. Co-cultures were treated with SHH-ligand (5 nM) and Hh inhibitors. Cell proliferation and expression of CD44/CD24 receptors were evaluated at 72 h. Significance was determined via Student’s *t*-test * *p*-value < 0.05, ** *p*-value < 0.01. (**E**) Proliferation of MDA-MB-468 in co-culture with TGFβ-Hs578t treated with SHH-ligand, cyclopamine (3 uM), and GANT61 (5 uM). Data represent mean +/− SEM of three to experiments with *n* = 4. Significance was determined via ANOVA analysis ** *p*-value < 0.01.

Recently, Hh signaling has been associated as a promoter of EMT [42,43], but its participation in tumor–stroma mediated growth was not examined. Tumor cells that undergo EMT are indistinguishable from fibroblasts in tissues due to their similarities in phenotypic markers, including SMA and VIM. We developed a co-culture model composed by MDA-MB-468 and Hs578t to examine the effect of EMT. Hs578t is a human TNBC cell line that undergoes EMT in response to TGF-β treatment (TGFβ-Hs578t). Main Hh signaling components and EMT markers were confirmed by positive immunostaining of SMA, VIM, primary cilium and PTCH1 receptor in Hs578t cells (Figure 5A,B). As expected, FAP protein, which is specific to fibroblasts, was not expressed in TGFβ-Hs578t cells. All Hh signaling transcripts were found in high levels relative to GAPDH. Only SMO was significantly overexpressed in response to exogenous addition of SHH-ligand, further confirming Hh signaling activity in TGFβ-Hs578t (Figure 5C). Co-culture studies show that TGFβ-Hs578t significantly enhanced the growth of MDA-MB-468 cells and the non-tumorigenic 184B5 cell line in response to SHH-ligand. However, this effect was not observed in cultures with Hs578t (Figure 5D), suggesting that phenotypic changes, associated to TGF-β treatment, impact the response to SHH-ligand at the secretome level. Similar behavior was observed in MDA-MB-231 co-cultures, but this growth effect was independent of exogenous addition of SHH-ligand, potentially due to the high levels of endogenous SHH-ligand expressed in this cell line. Exogenous addition of SHH-ligand did not stimulate the growth of TGFβ-Hs578t cells (Figure S8). All Hh inhibitors examined decrease tumor cell growth in co-cultures, but only the combination of cyclopamine and GANT61 was significant (Figure 5E). The results here confirm the participation of cells that undergo EMT as a therapeutic target of Hh inhibitors.

### 3.6. Adipose Mesenchymal Stem Cells Modulate Response to Hh Inhibitors

**Figure 6:**
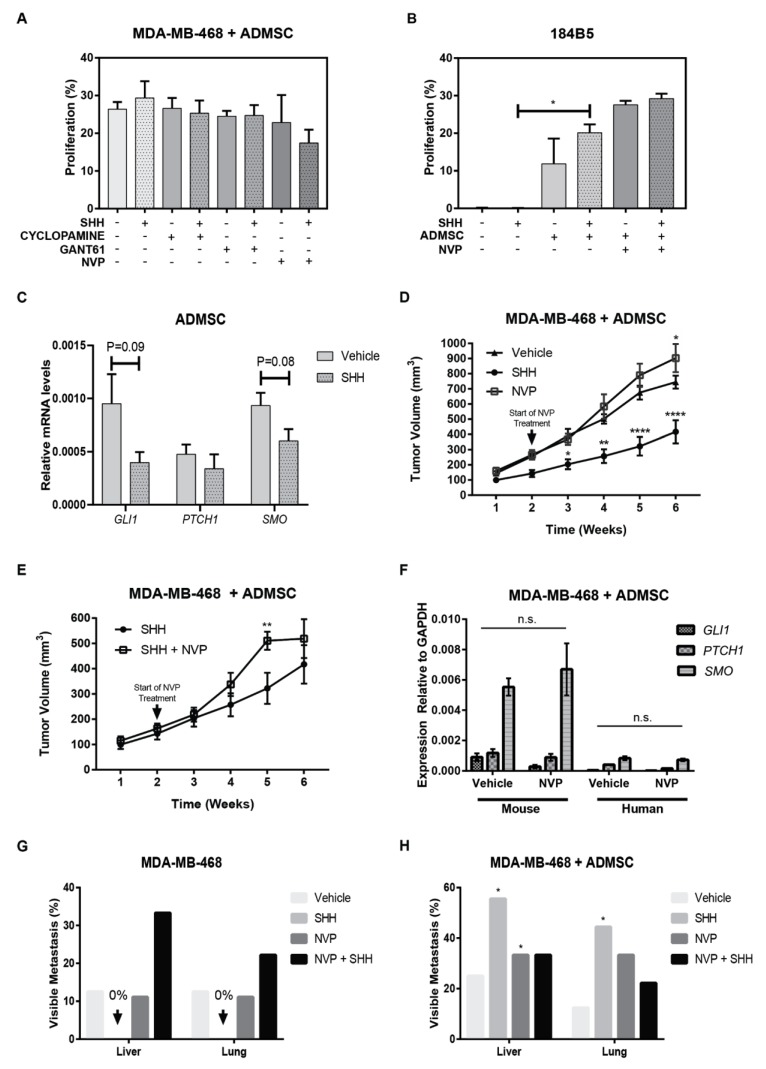
Mesenchymal cells modulate the effect of Hedgehog (Hh) signaling. (**A**,**B**) Proliferation analysis of 5-ethynyl-2’-deoxyuridine (EdU)+ MDA-MB-468 and 184B5 cells co-cultured with adipose mesenchymal stem cells (ADMSC) treated with Sonic hedgehog (SHH)-ligand and Hh inhibitors for 96 h. Significance was determined via Student’s *t*-test, * *p* < 0.05. (**C**) Expression levels of glioma associated oncogene family zinc finger 1 (*GLI1*), patched1 (*PTCH1*) and Smoothened (*SMO*) genes relative to *GAPDH* at 24 h post-treatment. (**D**,**E**) Tumor growth curves in xenografted tumors composed by MDA-MB-468 + ADMSC +/− SHH-ligand. Daily dosage with NVP or Vehicle during the last four weeks. Data show the average mean +/− SEM of nine mice per treatment. Significance was determined via ANOVA analysis comparing Vehicle (D) or SHH (E) with NVP, * *p* < 0.05, ** *p* < 0.01, **** *p* < 0.0001. **F**) Expression levels of *GLI1*, *PTCH1*, and *SMO* relative to *GAPDH* in xenografted tumors using human and mouse-specific primers. Data represent mean +/− SEM of six mice per condition. No significant differences were observed by Student’s *t*-test. (**G**,**H**) Percentage of mice with visible metastases. Significance was determined by Fisher’s exact test, * *p* < 0.05.

Adipose-derived mesenchymal stem cells (ADMSCs) are an abundant mesenchymal cell subtype in breast tissues [44] that express similar phenotypic markers as fibroblasts, but little is known about their contribution to paracrine Hh signaling. We examined Hh signaling in tumor-ADMSCs co-cultures. Normal ADMSCs derived from the abdominal cavity were selected due to shared similarities with ADMSCs derived from breast tissues [45], documented studies supporting their sensitivity to Hh signaling to block differentiation, and their clonogenic properties [46,47]. Expression of the primary cilium, PTCH1, and fibroblasts markers, FAP, SMA, and VIM, was examined before conducting co-culture studies (Figure S9). ADMSCs stained positive for all phenotypic markers examined, confirming the heterogeneous expression of these pathological markers among mesenchymal cell subtypes. In vitro co-cultures, independent of the addition of SHH-ligand or Hh inhibitors, showed that ADMSCs did not affect proliferation rates of MDA-MB-468 (Figure 6A). However, ADMSCs significantly stimulated the proliferation of the growth-arrested cell monolayer of non-tumorigenic 184B5 cells. This enhanced growth effect was further exacerbated in animals treated with NVP (Figure 6B). *GLI1*, *PTCH1*, and *SMO* transcripts were all detected in ADMSCs. The expression of these transcripts was reduced by the exogenous addition of SHH-ligand, but this effect was of marginal significance (Figure 6C). To determine whether the presence of ADMSC will alter therapeutic response to Hh inhibition in vivo, the proliferative effect of SHH-ligand and sensitivity to SMO antagonist NVP were examined in xenografted tumors composed by MDA-MB-468 + ADMSC. In agreement with in vitro observations, treatment with NVP resulted in significantly-enhanced tumor growth rates in the MDA-MB-468 + ADMSC group (Figure 6D,E). Hh target genes were not suppressed in the mouse stroma (Figure 6E), suggesting that ADMSCs decrease the sensitivity to NVP treatment. Although SHH-ligand appeared to inhibit tumor growth rates (Figure 6D), the number of visible metastases was significantly increased as compared to parallel studies in MDA-MB-468 xenografts (Figure 6G,H). Thus, SHH and NVP were tumor suppressors in the MDA-MB-468 group but tumor promoters in the MDA-MB-468 + ADMSC group. The enhanced growth effect caused by the presence of ADMSCs is probably associated to a remodeling of the tissue, yet it shows that therapeutic benefit of SMO inhibition can be perturbed by the composition of the tumor stroma, further highlighting the relevance of the tumor microenvironment in the therapeutic outcome.

## 4. Discussion

Despite increasing evidence indicating that the stroma is the main target of Hh inhibitors, most studies still rely on single tumor cell models to assess the pre-clinical potential of Hh therapies. Our work confirms the absence of a correlation between tumor cell viability and the suppression of the canonical modality of the Hh pathway in TNBC cells. Other studies have reported that pharmacological inhibition of SMO at doses of 10 µM or higher [10,48] inhibit the growth of a subset of breast cancer cell lines in vitro; however, the high doses required to affect tumor cell growth, as compared to those needed for the inhibition of the pathway in the stroma, suggests that the observed growth inhibition is due to off-target effects or inhibition of non-canonical Hh signaling. Such non-canonical Hh signaling mechanisms might be mediated through GLI2 transcriptional activity and the suppression of growth and survival mechanisms predominant in TNBC cells such as epidermal growth factor receptor (EGFR) [49] and phosphatidylinositol 3-kinase(PI3K-AKT)- mammalian target of rapamycin(mTOR) signaling [35,50].

Our studies support previous studies of TNBC [19,24] pointing to the tumor stroma as a mediator of the oncogenicity of the Hh signaling pathway and the target of Hh inhibitors. Both human breast fibroblasts and mouse fibroblasts (NIH3T3) showed a robust response to SHH-ligand that correlated with enhanced tumor cell growth. Pharmacological inhibition of the Hh pathway in the tumor-adjacent stroma correlated with reduced tumor growth rates and tumor stemness in both culture and xenograft models, recapitulating some of the main pathological features reported, which supports the physiological relevance of the in vitro model. Thus, the evaluation of the therapeutic sensitivity to Hh inhibitors should include the examination of a tumor–fibroblast model, given that single tumor cell models can be misleading and less predictive of a physiological response.

The evaluation of culture parameters is important for the generation of in vitro models. Oftentimes, basic culture parameters such as cell number ratio or tumor–stroma proximity can seem trivial or unimportant in comparison to other experimental variables; however, they can have a significant influence on the interpretation of results. In this work, tumor growth rates were enhanced in both mixed and adjacent culture modalities, but tumor stemness was only enhanced in mixed cultures. This finding suggests that diffusible secreted factors stimulate bulk tumor growth, whereas cancer cell stemness is supported by cell–cell interactions likely driven by the extracellular matrix at tissue regions confined to the tumor–stroma interface. This idea is supported by studies from Cazet et al., in which active Hh signaling correlated with enhanced tumor growth and stemness in regions of abundant collagen I matrix found near areas of proximity between tumor and CAFs in both PDX models and patient samples of TNBC [24]. Such a localized effect of the Hh pathway in tumor stemness resembles the process observed during developmental stages of tissues. During embryonic development, a stable long-range gradient of SHH-ligand is established to specify neural progenitors and individual cell identities, resulting in the final neural pattern [51]. SHH-ligand is restricted by a negative feedback loop and is dependent on ligand lipidation. The lipidation probably restricts the location of the SHH-ligand to the extracellular membrane, minimizing its free-soluble form. In fact, our staining of SHH-ligand confirmed its presence and abundance in the extracellular membrane of TNBC cell lines, probably restricting its diffusivity in the examined adjacent culture modalities, as noted by the lack of transcriptional activity of the Hh pathway.

One interesting observation that resulted from our xenograft studies was the possibility that SHH-ligand may have a growth-suppressive role. Tumor growth volume was significantly reduced and Ki67 levels were decreased to levels below those achieved with NVP treatment in tumor xenografts composed by MDA-MB-468 single cells. A similar growth-suppressive effect was observed in single cultures of non-tumorigenic breast cell lines supplemented with SHH-ligand. We believe that this growth-suppressive effect is mediated by an excess in the soluble form of SHH-ligand that stimulates the secretion of growth-suppressive factors and/or intracellular apoptotic mechanisms. In our tumor xenograft model, the free-soluble form of SHH-ligand was released from beads co-injected with tumor cells. The abundance of the soluble form of SHH-ligand likely reached tissue regions beyond the tumor–stroma interface, leading to the activation of growth suppression mechanisms in the native epithelium or stroma of the mouse mammary gland. Further studies that evaluate the impact of the concentration and distribution of SHH-ligand on cell growth will be needed to determine its potential to counteract tumor growth, as the current role of SHH-ligand in adult breast tissues remains largely speculative.

Beyond tumor growth and CSC expansion, the Hh pathway is associated with induction of EMT in tumor models of breast cancer as well as other tissues [23,52]. Our data show that tumor cells that undergo EMT are responsive to Hh signaling and support enhanced tumor growth rates at similar levels to those observed with fibroblasts. This could have important implications in the pathology of Hh signaling and the selection of a therapeutic regimen. For example, in tissues in which Hh signaling is driven primarily by cells that undergo EMT, matrix-driven tumor stemness is likely to be absent as fibroblasts are known to actively remodel the collagen I matrix at the tumor interface, whereas cells that undergo EMT have limited activity [53]. Given the importance of GLI1 in the Hh pathway on promoting EMT, inhibition of GLI1 rather than SMO is likely a better therapeutic option for suppressing Hh signaling in TNBC. In our in vitro models, both fibroblasts and EMT co-cultures were sensitive to the GLI1 antagonist GANT61, and this therapeutic response was more robust than SMO inhibitors alone. This sensitivity to GLI1 antagonist is in agreement with previous reports indicating that targeting of GLI1/2 function through GANT58 and GANT61 decreased tumor growth of prostate [54] and breast cells [52,55]. Another benefit of abolishing the function of GLI proteins is dual targeting of oncogenic signals in other pathways shown to cross-talk with Hh including TGF-β and wingless–related integration site (WNT) [55,56].

The inclusion and selection of the stroma are important for the tissue-mimetic predictive capability of an in vitro model and optimization of therapeutic strategies. The source of the Hh-responsive stroma is presumed to be CAFs only, yet molecular markers used to identify these cells in tissues (e.g., smooth muscle actin) are shared among other mesenchymal cell subtypes abundant in breast tissues, such as the cells shown herein, EMT, normal fibroblasts, and ADMSCs. Our studies demonstrated that normal fibroblasts and tumor cells that undergo EMT can support tumor cell growth in a Hh-dependent manner at similar levels to those observed in CAFs. Yet, the precise mechanisms downstream of Hh signaling in each tumor–mesenchymal model were not part of the scope of this study and remain undetermined. Further mechanistic studies will be needed to determine whether such downstream mechanisms are similar or differ among mesenchymal cell subtypes supportive of the oncogenicity of the Hh pathway. The identification of such mechanisms will be of importance for targeting mesenchymal subtypes in tissues and the optimization of therapeutic regimens in a patient-specific manner.

## 5. Conclusions

Overall, the studies presented provide a tumor–mesenchymal in vitro model that supports the tumorigenicity of the Hh pathway reported in tumors of the TNBC subtype. Our studies show that cells of a mesenchymal phenotype can support Hh-driven tumor growth and stemness but can also perturb therapeutic sensitivity to Hh inhibitors in TNBC. Thus, tumor–mesenchymal models, such as those developed in this work, will be of great value for the identification of the mechanisms and oncogenic targets associated with a mesenchymal cell subtype. In future studies, we will build on this tumor–stroma model to transition from a 2D and 3D model and incorporate organotypic models for boosted biomimetic capabilities. The examination of the paracrine modality of the Hh signaling pathway in pre-clinical models will provide enhanced biological complexity for improved assessment of the efficacy of therapeutic targets and the identification of new strategies targeted to the stroma.

## Supplementary Materials

The following are available online at https://www.mdpi.com/2072-6694/11/10/1522/s1, Figure S1: Full western blots of breast cancer cells, Figure S2: Full western blot of fibroblast cell lines and MCF10A Figure S3: Effect of SHH-ligand and Hh inhibitors in Luminal A and non-tumorigenic breast cancer cells, Figure S4: Characterization of mesenchymal markers and Hh signaling in fibroblasts, Figure S5: Primary cilium in fibroblasts, Figure S6: Proliferation of ER+ tumor cells in co-culture with HMF, Figure S7: Hh signaling in NIH3T3 across culture modalities, Figure S8: Proliferation of NIH3T3 and Hs578t cells in tumor co-cultures, Figure S9: Characterization of Hh components in ADMSC.
