# Supplementary material for: Mesenchymal Cells Support the Oncogenicity and Therapeutic Response of the Hedgehog Pathway in Triple-Negative Breast Cancer
Source: Cancers (Basel). 2019 Oct 10;11(10):1522. doi: 10.3390/cancers11101522 (PMC6826628; doi:10.3390/cancers11101522)
Supplement: Supplementary file 1 [file cancers-11-01522-s001.pdf]

Supplementary Figures

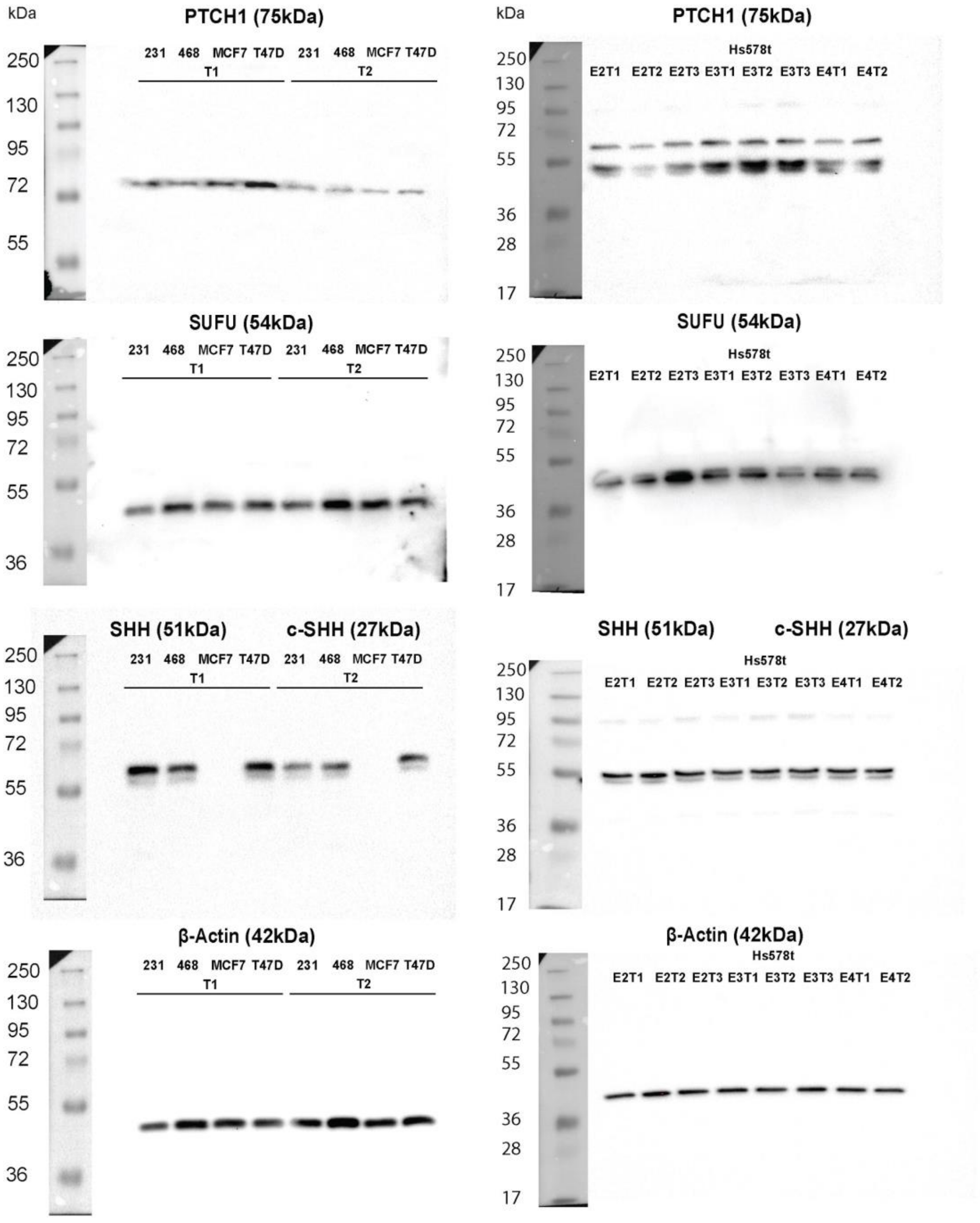

Figure S1. Full western blots of breast cancer cells. Whole western blots to detect PTCH1 receptor (75kDa), full-length (51kDa) and c-product subunit (27 kDa) of human SHH-ligand and SUFU (54 kDa) in MDA-MB-231, MDA-MB-468, MCF7, Hs578t and T47D cell lines. β-actin (42 kDa) was used as a loading control. The same gel was stained several times to image each protein individually. The molecular weight ladder and protein bands were imaged in color and chemiluminescence respectively. The color image (ladder) was separated from the chemiluminescent image to improve the clarity of the protein bands.

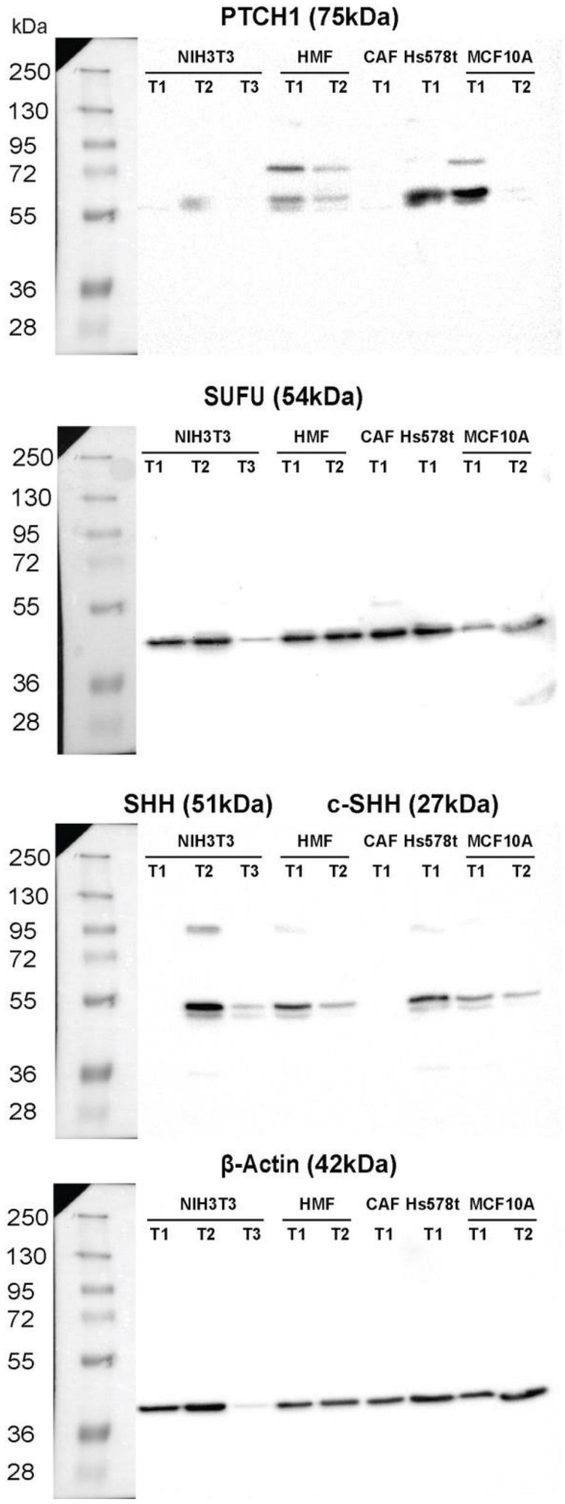

Figure S2. Full western blot of fibroblast cell lines and MCF10A. A full western blot of samples (T) examined to detect protein bands for PTCH1 receptor (75kDa), full-length (51kDa) and c-product subunit (27 kDa) of SHH-ligand and SUFU (54kDa) in NIH3T3 (T1–T3), HMF (T1–T2), Hs578t (T1), CAF (T1) and MCF10A (T1–T2) cell lines. β-actin (42 kDa) was used as a loading control. The same gel was stained several times to obtain a blot image of each protein. The molecular weight ladder was imaged in color and bands in

chemiluminescence. The color image (ladder) was unmerged from the chemiluminescent image to increase the clarity of the protein bands.

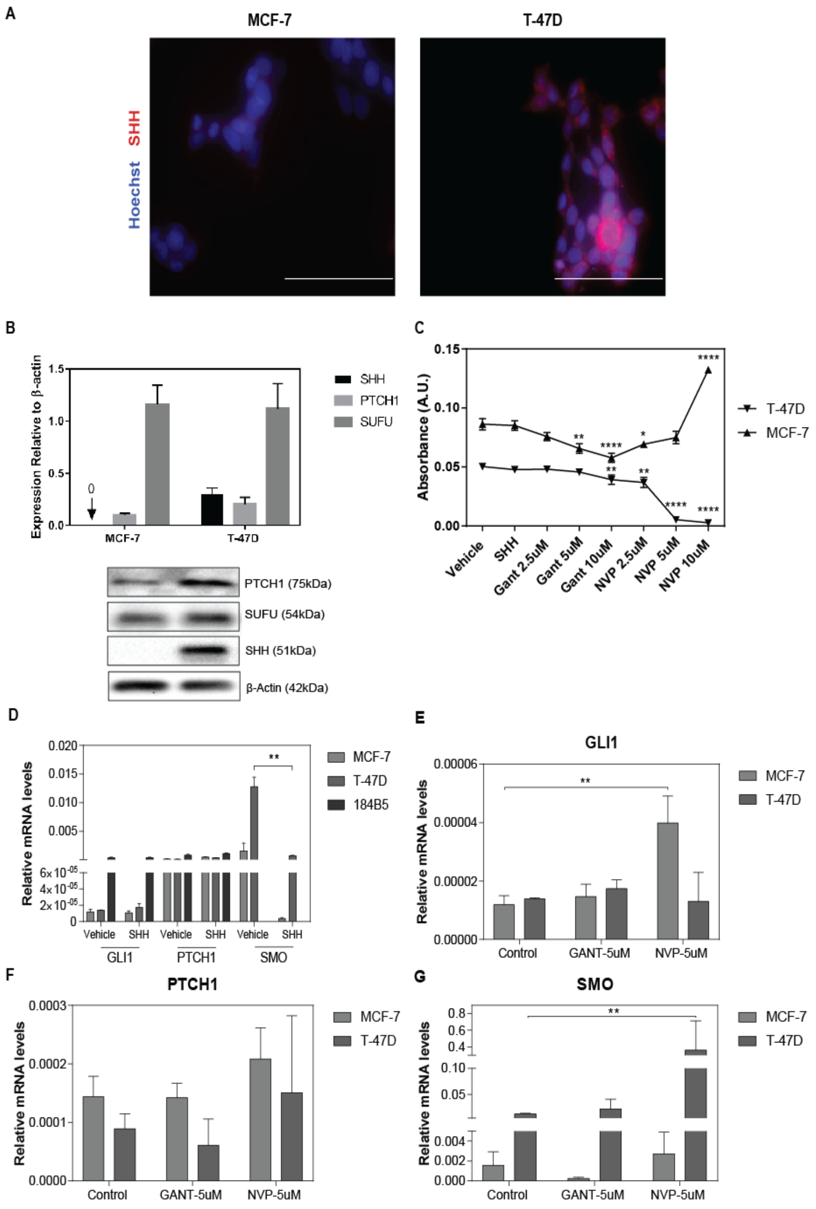

Figure S3. Effect of SHH-ligand and Hh inhibitors in Luminal A and non-tumorigenic breast cancer cells. **A**) Immunofluorescent staining of SHH expression in MCF-7 and T-47D cells. SHH (Red) and Hoechst (Blue). Scale bar = 100 µm. **B**) Expression of PTCH1 receptor (75 kDa), SUFU (54kDa) and SHH-ligand (51 kDa), in tumor cells relative to β-actin (42 kDa). Data represent the relative mean intensity ± SEM of 3-4 independent experiments. Samples were distributed among 2-3 blots (see supplementary figure S8). Representative protein bands are shown. **C**) Tumor cell viability was evaluated in response to 5nM SHH-ligand and 2.5, 5 and 10 µM concentrations of NVP and GANT61 (GANT) for 96 hrs using the XTT assay. Data represent mean ± SEM of 3 independent experiments with $n = 4$. Significance was determined via ANOVA analysis * p-value < 0.05, ** p-value < 0.01, **** p-value < 0.0001. **D–G**) Expression levels of *GLI1*,

PTCH1 and *SMO* genes relative to *GAPDH* exposed to exogenous SHH-ligand for 24hrs (**D**) and Hh inhibitors, GANT (5µM) and NVP (5µM) for 48hrs (**E**–**G**). Data represent mean ± SEM of 3-4 experiments with n = 3–4. Significance was determined via ANOVA analysis ** p-value < 0.01.

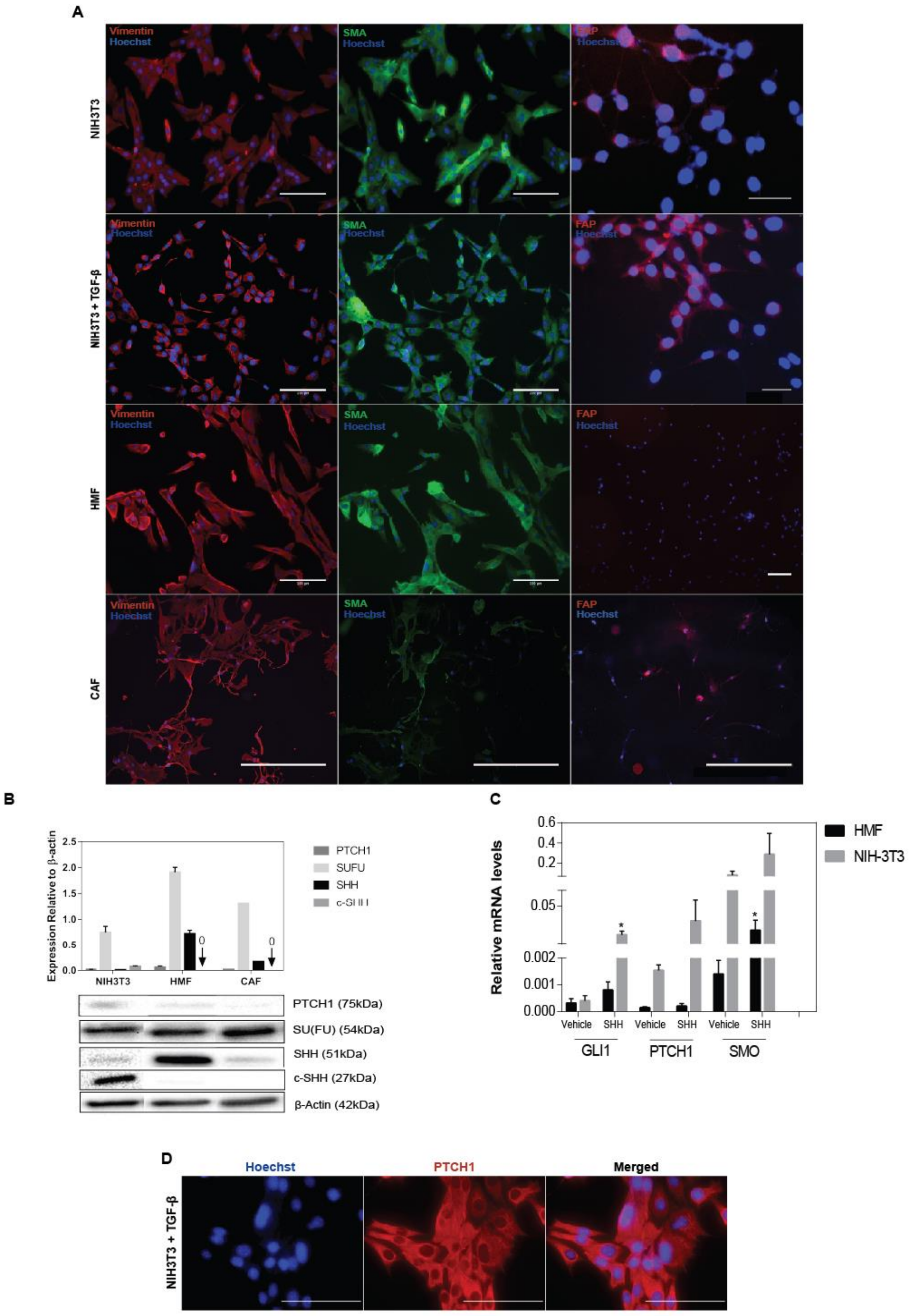

Figure S4. Characterization of mesenchymal markers and Hh signaling in fibroblasts. **A**) Expression of SMA, FAP and Vimentin was evaluated by immunostaining. The color contrast was enhanced digitally for

improving contrast. The color blue in all the images represents Hoechst nuclear staining. Scale bar = 100 μm for NIH3T3, TGF-β treated NIH3T3 and HMF. Scale bar = 200 μm for CAFs. **B**) Expression of PTCH1 receptor (75 kDa), SUFU (54 kDa), full-length (51 kDa) and c-product subunit (27 kDa) of human SHH-ligand were quantified relative to β-actin (42 kDa). Data represents the relative mean intensity ± SEM of 3 independent experiments with n = 3 except for CAF which had 1 experiment. Representative protein bands are shown (see supplementary figure S9). **C**) NIH3T3, HMF, and CAF cells treated with SHH-ligand (5nM) for 24hrs. Expression of levels of *GLI1*, *PTCH1* and *SMO* were quantified relative to *GAPDH*. Data represent the mean ± SEM of 3 independent experiments with n = 4. Student's T-test was used for comparisons between two data sets, * $p < 0.05$. **D**) Staining of Patched 1 (PTCH1) membrane receptor (red) and nuclear Hoechst (blue) on TGF-β treated NIH3T3. Images were digitally enhanced using Fiji for improving contrast. Scale bar = 100 μm.

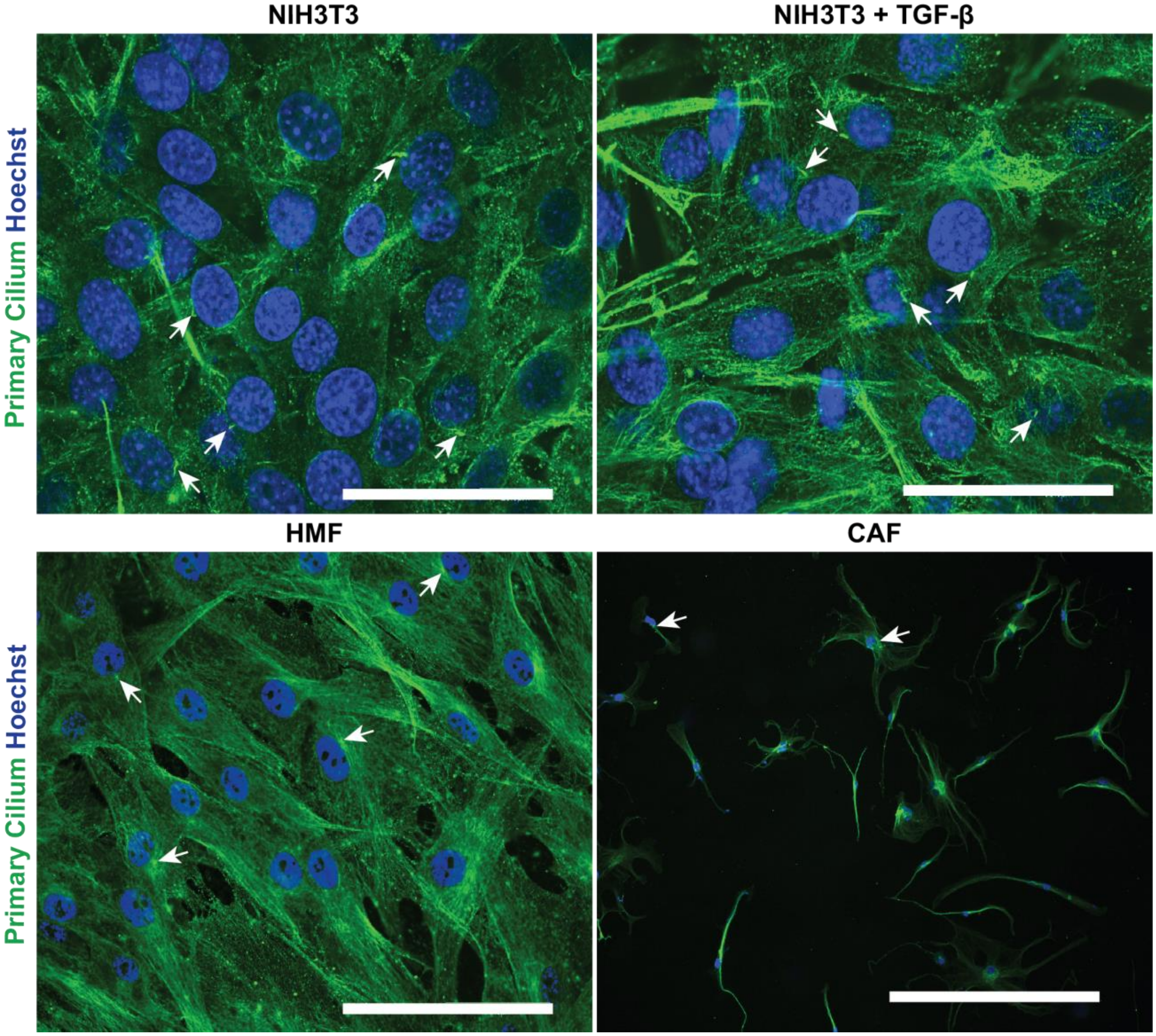

Figure S5. Primary cilium in fibroblasts. Expression of acetylated-alpha tubulin (primary cilium) (green) as evaluated by immunostaining and confocal microscopy. White arrows point to the primary cilium staining. The color blue in all the images represents nuclear staining using Hoechst. Scale bar = 100 μm for NIH3T3, NIH3T3 + TGF-β and HMF. Scale bar = 200 μm for CAF.

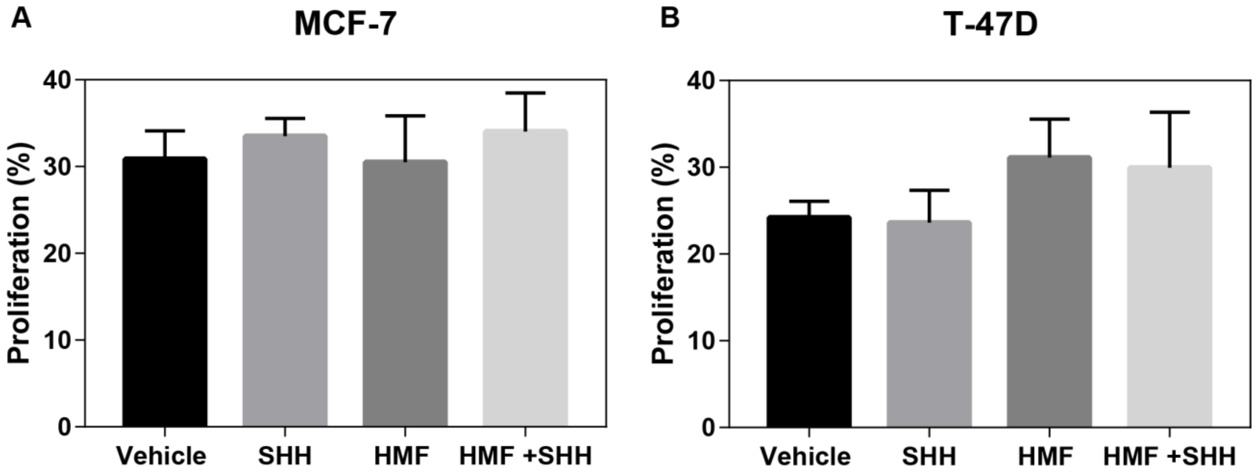

Figure S6. Proliferation of ER+ tumor cells in co-culture with HMFs. **A**) Proliferation of MCF-7 cells in co-cultures with HMF at 96 hours, treated with SHH ligand **B**) Proliferation of T-47D in co-cultures with HMF at 96 hours, treated with SHH ligand. Two-way ANOVA was used for statistical comparison and determination of significance. Data represent the mean ± SEM of 3-5 independent experiments with $n = 4$. No significant differences were observed.

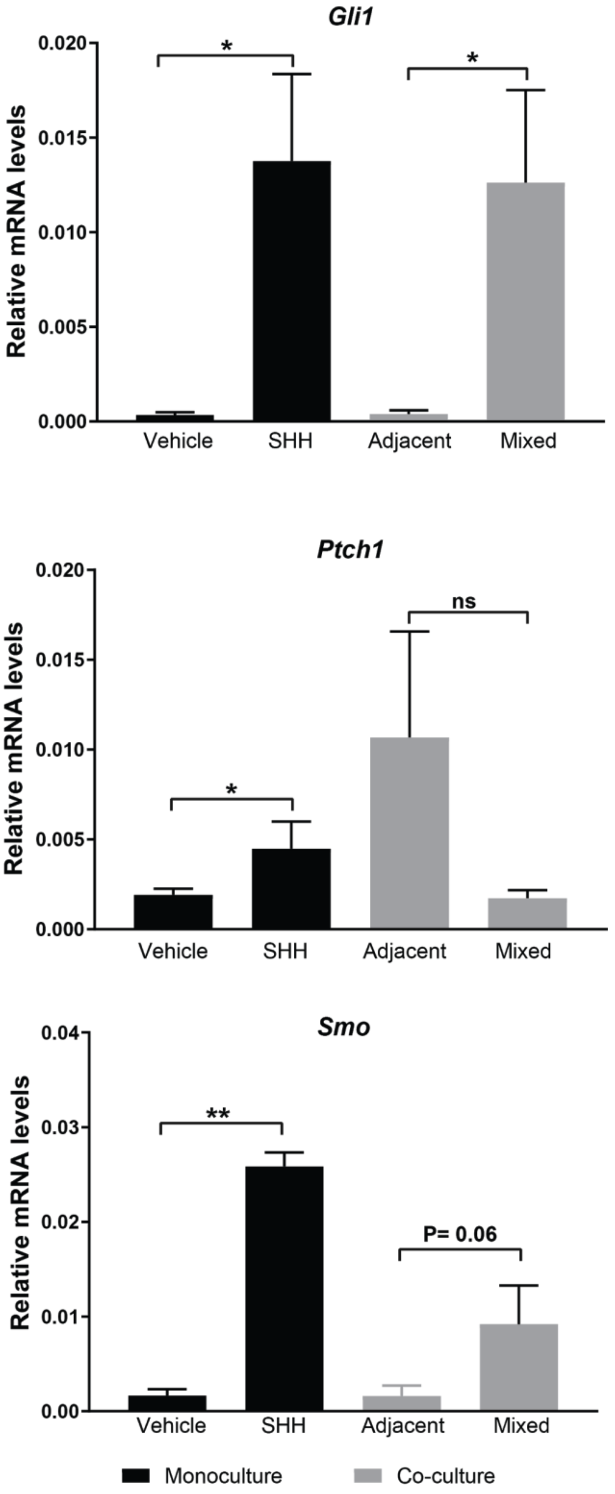

Figure S7. Hh signaling in NIH3T3 across culture modalities. NIH3T3 were cultured as a single cell monolayer (monoculture) or with GFP-tagged MDA-MB-231 (co-culture) for 96hrs. Monocultures were treated with SHH-ligand (5nM) for 96hrs. Expression of levels of murine *Gli1*, *Ptch1* and *Smo* were quantified relative to *Gapdh*. Data represent the average mean ± SEM of 3 independent experiments with n = 3-6. Significance determined by Student t-test * p-value < 0.05 and ** p-value < 0.01.

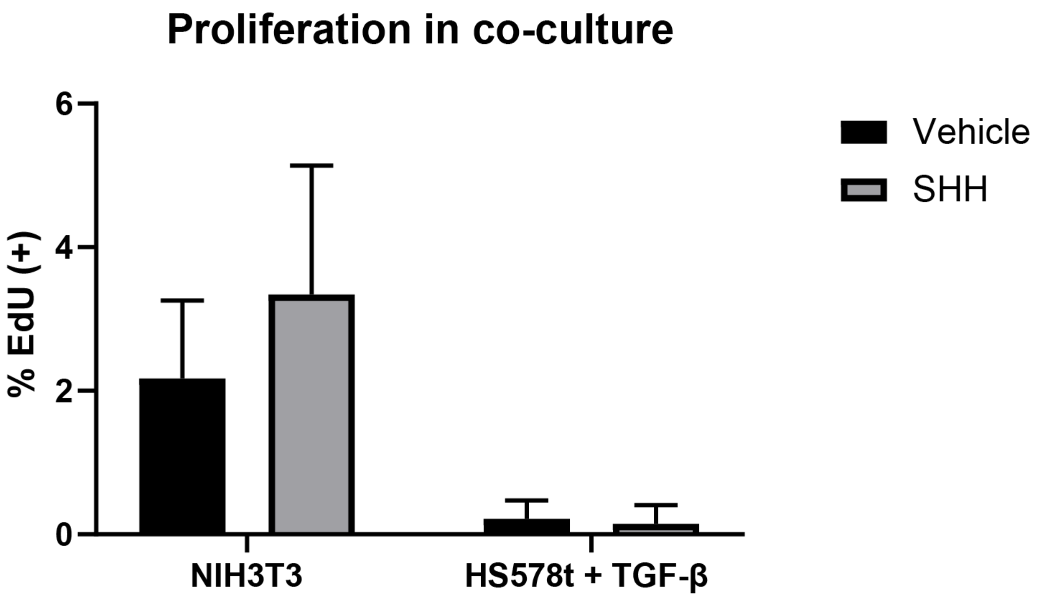

Figure S8. Proliferation of NIH3T3 and Hs578t cells in tumor co-cultures. Percentage of EdU (+) cells post-treatment with 5nM SHH-ligand or Vehicle in co-culture with MDA-MB-468 at 96 hours. Data shown represent the mean ± SD of 2 independent experiments with *n* = 3–18. No significant differences were observed.

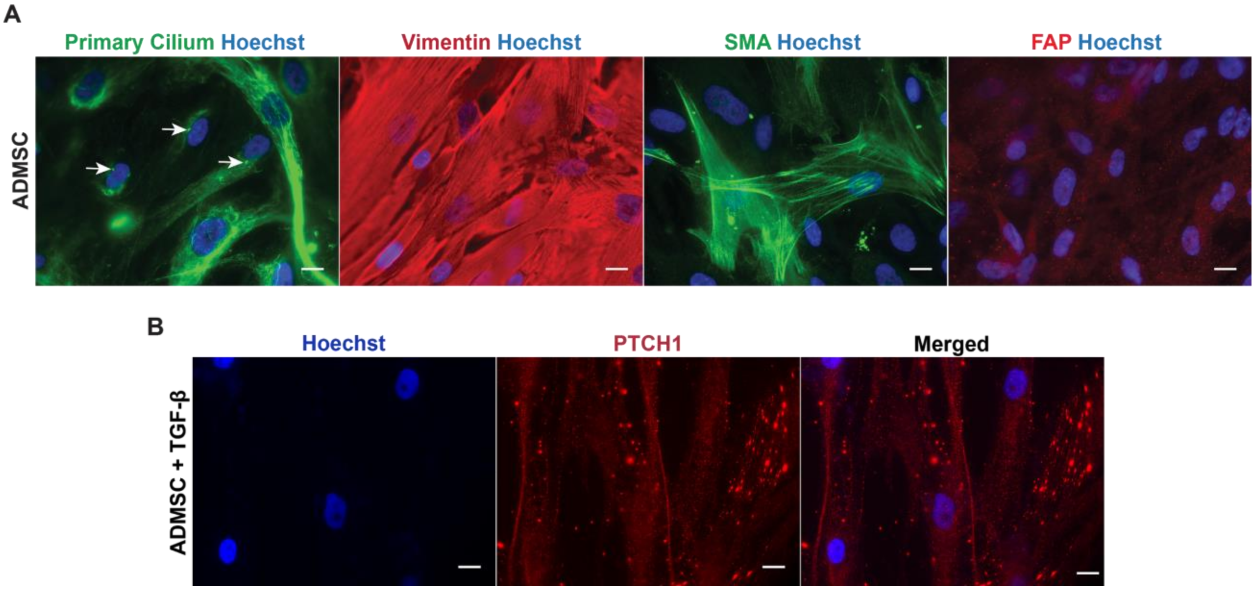

Figure S9. Characterization of Hh components in ADMSC. **A**) Staining of mesenchymal markers acetylated-alpha tubulin (primary cilium), Vimentin, alpha-smooth muscle Actin (SMA) and Fibroblast activation protein (FAP). White arrows indicate sample primary cilium staining. Images were digitally enhanced using Fiji for better visualization of mesenchymal markers. Scale bar = 10 μm. **B**) Staining of Patched 1 (PTCH1) membrane receptor (red) and nuclear Hoechst (blue). The color blue in all the images represents nuclear staining using Hoechst. Images were digitally enhanced using Fiji for improving contrast. Scale bar = 10 μm.
